# Two sequential gene expression programs bridged by cell division support long-distance collective cell migration

**DOI:** 10.1242/dev.202262

**Published:** 2024-05-17

**Authors:** Jingjing Sun, Ayse Damla Durmaz, Aswini Babu, Frank Macabenta, Angelike Stathopoulos

**Affiliations:** ^1^ California Institute of Technology, Division of Biology and Biological Engineering, 1200 East California Boulevard, Pasadena, CA 91125, USA; ^2^ Faculty of Biology, Ludwig-Maximilians Universität München, München, 82152 DE, Germany; ^3^ California State University, Monterey Bay, Seaside, CA 93955, USA

**Keywords:** Collective cell migration, Transcription programs, Cell cycle progression, Spatiotemporal gene expression, Caudal visceral mesoderm

## Abstract

The precise assembly of tissues and organs relies on spatiotemporal regulation of gene expression to coordinate the collective behavior of cells. In *Drosophila* embryos, the midgut musculature is formed through collective migration of caudal visceral mesoderm (CVM) cells, but how gene expression changes as cells migrate is not well understood. Here, we have focused on ten genes expressed in the CVM and the cis-regulatory sequences controlling their expression. Although some genes are continuously expressed, others are expressed only early or late during migration. Late expression relates to cell cycle progression, as driving *string/Cdc25* causes earlier division of CVM cells and accelerates the transition to late gene expression. In particular, we found that the cell cycle effector transcription factor E2F1 is a required input for the late gene *CG5080*. Furthermore, whereas late genes are broadly expressed in all CVM cells, early gene transcripts are polarized to the anterior or posterior ends of the migrating collective. We show this polarization requires transcription factors Snail, Zfh1 and Dorsocross. Collectively, these results identify two sequential gene expression programs bridged by cell division that support long-distance directional migration of CVM cells.

## Teaser

**Highlighted Article:** Insights into how transition of transcriptional states is coordinated along with cell cycle progression during long-distance migration of cell collectives.

## INTRODUCTION

Collective cell behavior is controlled by a variety of different cues, many of which intersect in a multitude of elegant ways, to promote precise morphogenetic events such as forward motility, proliferation and cell death (Lecuit and Le Goff, 2007). Dysregulation of any of these processes can have serious consequences, including abnormal function and the development of diseases such as cancer (Friedl and Gilmour, 2009; Letai, 2017). The regulation of morphogenetic programs, such as collective cell migration, often involves the interpretation of extracellular signals. Dynamic gene expression has been associated with various stages of cell migration for individual cells as well as collectives, and likely controls the changing morphological behaviors associated with cells as they accomplish long-distance migrations (e.g. Busch et al., 2008; Schwarz et al., 2012; Morrison et al., 2021). Studying how this dynamic gene expression is regulated may provide insight into the inputs cells use to navigate their changing environments.

In *Drosophila,* the caudal visceral mesoderm (CVM) cells undergo a long-distance, directional migration from posterior to anterior in order to position themselves along the entire length of the developing gut and ultimately help form the longitudinal muscles of the gut (Sun et al., 2020). CVM cell migration takes ∼6 h, one quarter of *Drosophila* embryogenesis (∼24 h); therefore, the microenvironment changes drastically over the course of their migration as the embryonic tissues diversify in complexity. CVM cells originate at the ventroposterior end of the cellular blastoderm embryo (stage 5), through the combined input of mesodermal gene *snail* (*sna*) and terminal genes *brachyenteron* (*byn*) and *forkhead* (*fkh*), but not requiring the mesodermal gene *twist* (Kusch and Reuter, 1999). At stage 10, after germband extension, the CVM cells initiate synchronous bilateral migration. First, cells move along the posterior midgut (PMG) before traveling along the trunk visceral mesoderm (TVM) cells, which serve as the substrate track (Macabenta and Stathopoulos, 2019). Transcription factors (TFs) HLH54F, Byn, Fkh and Zinc-finger homeodomain 1 (Zfh1) are required for normal migration of the CVM cells (Kusch and Reuter, 1999; Ismat et al., 2010). Although fibroblast growth factor (FGF) signaling is known to play a role, as FGF mutant CVM cells appear to veer away from the TVM substratum and ultimately undergo apoptosis (Mandal et al., 2004; Kadam et al., 2012), little is known about how these mesodermal TFs coordinate to control migration or what extracellular cues are influential.

About halfway through their migration, the CVM cells undergo cell cycle-dependent quality control that involves intersecting signals by FGF and bone morphogenetic protein (BMP) (Macabenta et al., 2022). During anteriorward migration, the CVM cells encounter and process secreted Decapentaplegic (Dpp) ligand, the *Drosophila* BMP2/4 homolog. Dpp initiates G2-M cell cycle progression in the CVM cells, which results in coordinated cell division at stage 11/12. Additionally, this G2-M transition promotes the upregulation of the cell death gene *head involution defective* (*hid*) in all CVM cells, which allows for the rapid elimination of any cells that have wandered away from the TVM substrate. FGF signaling through the CVM-expressed FGF receptor Heartless, which is activated by FGF ligands expressed in the TVM, directly antagonizes pro-apoptotic Hid protein, which ensures the survival of cells that have remained on-track (on the TVM) but promotes death for those that have wandered off-track. Although this previous study demonstrated that balanced input by FGF and BMP signaling is important to support cell survival and proliferation, how cells modulate their gene expression programs before and after this quality control decision remains poorly understood.

In attempts to gain additional insight into regulatory mechanisms affecting CVM cell migration, we obtained transcriptomic information using either RNA sequencing of an enriched CVM cell sample (Bae et al., 2017) or single-cell analysis of embryos at the gastrulation stage (Sun et al., 2023). A number of genes have been identified that are enriched within the CVM at stages 10-11. Surprisingly, gene expression was retained in FGF mutants for all those examined (Bae et al., 2017). However, given our finding that expression of *hid* in CVM cells is regulated by the cell cycle (Macabenta et al., 2022), we hypothesized that the cell cycle may more broadly influence gene expression in these cells. Additionally, analysis of our recent single-cell RNA-seq dataset showed that expression within the CVM migrating collective can be polarized, with genes *kon-tiki* (*kon*) and *Dorsocross 2* (*Doc2*) enriched in anterior cells and the gene *Grip* enriched in posterior cells (Sun et al., 2023). In this study, we describe how spatial and temporal gene expression is controlled within this migrating collective through analysis of enhancer sequences and TF inputs, and by further investigating the role of the cell cycle.

## RESULTS

### Identification of enhancers that drive gene expression in migrating CVM cells

**Fig. 1.**
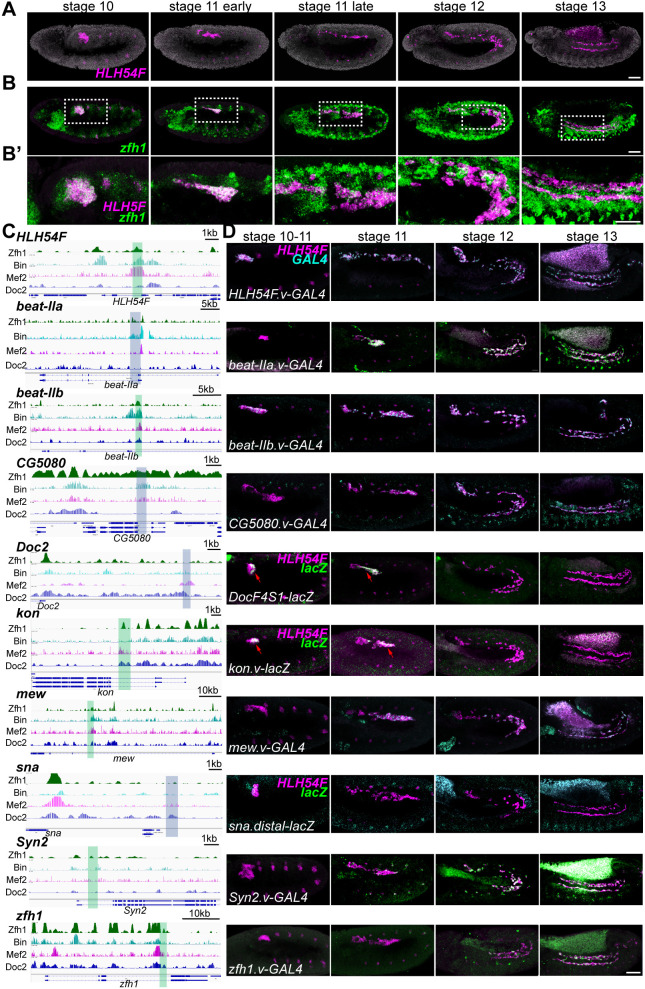
**Enhancers supporting expression in CVM cells exhibit spatiotemporal differences in gene expression outputs.** (A-B′) *Drosophila* embryo time series (stage 10-13) showing expression detected by *in situ* hybridization using a hybridization chain reaction (HCR) probe to *HLH54F* transcript (magenta) either alone (A) or when colocalized relative to *zfh1* (green; B). B′ is a magnified view of CVM cells outlined in B. Anterior to the left and dorsal upwards. (C) ChIP data for the transcription factors Zfh1, Bin, Mef2 and Doc2. Regions assayed for enhancer activity are highlighted in green (Zfh1-binding enriched) or blue (Zfh1-binding not enriched). (D) Reporter outputs for indicated enhancer regions (C) using *in situ* hybridization with regular riboprobes to *GAL4* and *HLH54F* or with HCR probes to *lacZ* and *HLH54F*. Scale bars: 50 μm.

To understand how gene expression is regulated as CVM cells migrate, we sought to characterize the TFs that are continuously expressed by CVM cells during stages 11-13. Byn, Fkh and Sna TFs regulate CVM specification at stage 5 by controlling the localization of *HLH54F* expression in a ventroposterior region of the cellular blastoderm embryo, but are no longer expressed in the CVM following stage 10 (germband elongated embryo) (Ip et al., 1994; Kusch and Reuter, 1999; Ismat et al., 2010). CVM cells continuously express *HLH54F* throughout their migration (Fig. 1A; Ismat et al., 2010). Zfh1, the *Drosophila* homolog of ZEB, is a putative transcriptional repressor that contains zinc fingers and homeodomains (Postigo et al., 1999), and is expressed in CVM cells from stage 5 onwards, including stage 13 (Broihier et al., 1998; Bae et al., 2017). However, at stage 12, *zfh1* transcripts are barely detectable, but protein products are present in nuclei at low levels (Fig. S1A,B). Both *HLH54F* and *zfh1* play important roles in regulating gene expression within the CVM cells and are required for their migration, survival and differentiation (Ismat et al., 2010; Bae et al., 2017).

We used available ChIP-seq data for Zfh1 and several other TFs expressed in the CVM. Biniou (Bin) is the sole *Drosophila* representative of the FoxF family of forkhead domain genes and has a key role in the specification of visceral mesoderm and its derived gut musculature (Zaffran et al., 2001). Mef2 is a MADS-box TF with a general role in gene expression in muscles, including the visceral mesoderm (Lilly et al., 1995). Dorsocross 2 (Doc2) is one of three T-box TFs (Doc1, Doc2 and Doc3) encoded by the Dorsocross cluster; Doc2 and Doc1 paralogs are both expressed in the CVM (Reim et al., 2003; Ismat et al., 2010; Sun et al., 2023). Using the available ChIP data relating to these four TFs (i.e. Zfh1, Bin, Mef2 and Doc2; Jakobsen et al., 2007; Zinzen et al., 2009; Junion et al., 2012; Wu et al., 2019), we scanned for their co-occupancy to DNA sequences flanking 16 genes that had previously been confirmed to be expressed in actively migrating CVM cells during stages 10-13 (Bae et al., 2017). Enhancers regulating *HLH54F* and *Doc2* had been identified previously (Ismat et al., 2010; Kadam et al., 2012). We found Zfh1, Bin, Mef2 and/or Doc2 ChIP-occupied regions corresponding to these published enhancer sequences (Fig. 1C: *HLH54F* and *Doc2*). Moreover, the ChIP binding within the *HLH54F* intron identified a more minimal enhancer sequence of 653 bp able to support reporter expression in the CVM (Fig. 1D).

Taking a similar approach, we also scanned genomic sequences in the vicinity of the other 14 genes for occupancy of these factors Zfh1, Bin, Mef2 and Doc2 to flanking non-coding sequences (Fig. 1C). Putative enhancer regions were tested through reporter assay (see Materials and Methods), and eight additional enhancers were identified supporting expression related to the following genes: *beaten path IIa* (*beat-IIa*), *beaten path IIb* (*beatII-b*), *CG5080*, *kon-tiki* (*kon*), *multiple edematous wings* (*mew*), *snail* (*sna*), *Syntrophin-like 2* (*Syn2*) and *zfh1*. Expression associated with each reporter (i.e. *Gal4* or *lacZ*) was colocalized with *HLH54F* using *in situ* hybridization in fixed embryos at stages 10-13, confirming their expression in CVM cells (Fig. 1D; see Materials and Methods). A subset failed to support expression (Fig. S1C). Of the ten enhancer sequences that support expression in the CVM, six are associated with a clear Zfh1-binding peak (i.e. *HLH54F*, *beat-IIb*, *kon*, *mew*, *Syn2* and *zfh1*) and four are not (i.e. *beat-IIa*, *CG5080*, *Doc2* and *sna*) (Fig. 1C). We postulate that for those not bound by Zfh1, HLH54F is the pivotal input to maintain CVM cell identity, as Zfh1 has been suggested to carry out a parallel function by supporting cell migration (Ismat et al., 2010).

### Characterization of dynamic gene expression within CVM cells and comparison to enhancer-supported expression

**Fig. 2.**
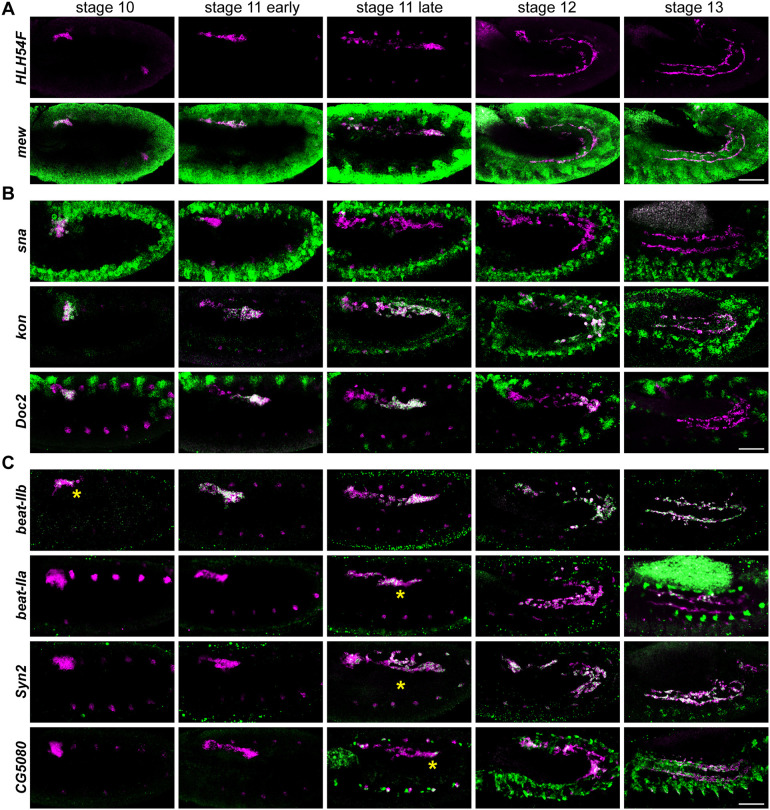
**Characterization of genes expressed in migrating CVM cells.** (A-C) CVM-specific gene expression facilitated by colocalization of transcripts using hybridization chain reaction probes for indicated genes (green) and *HLH54F* (magenta) through *in situ* hybridization. Genes are either continuously expressed (A), expressed predominantly early but become restricted (B) or expressed late (C). Asterisks in C indicate initiation of late gene expression. Anterior to the left and dorsal upwards. Scale bars: 50 μm.

**Fig. 3.**
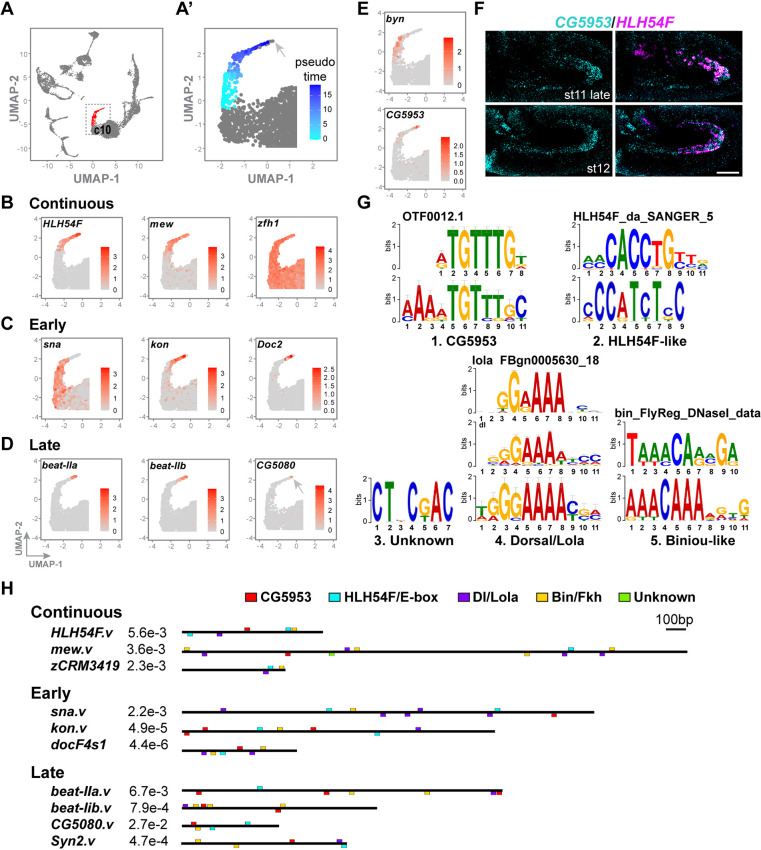
**scRNA-seq data confirm gene expression dynamics; computational analysis of enhancer sequences identifies important inputs.** (A-D) UMAP plots of single-cell transcriptomic data relating to embryos predominantly of stages 7-11 from a published study (Sun et al., 2023) in which CVM cells are contained in one particular cluster 10 (c10, red; A). (A′) Focused view of the UMAP plot of c10 cells (area outlined in A) analyzed by monocle pseudotime analysis in which younger versus older cells are colored in cyan and dark blue, respectively. Expression of the three categories of indicated CVM-expressed genes in c10 with red color intensity indicating the relative levels of gene expression (B-D). (E) UMAP plot showing *byn* and *CG5953* expression suggesting that, although *byn* is expressed early, *CG5953* is expressed late in the CVM (E, compare with C and D). (F) Hybridization chain reaction *in situ* hybridization confirms the CVM-specific expression of *CG5953* at stage 11-12 (cyan) relative to *HLH54F* (magenta, marking CVM cells). (G) *De novo* PWM logos identified via XSTREME with comparisons to published motifs; *E-*value=6e-005 for each motif. (H) Locations of identified consensus binding motifs (*de novo* motifs from G) on identified enhancers generated by MAST. Block diagrams of enhancers named according to the genes they regulate; zCRM3419 relates to the *zfh1.v* enhancer that supports the late gene expression (see Fig. 1D). *E* value for each corresponding enhancer sequence is shown on the left. Anterior to the left and dorsal upwards. Scale bar: 50 μm.

To fully capture the spatiotemporal gene expression patterns associated with these ten genes, we performed *in situ* hybridization with more specific and sensitive hybridization chain reaction (HCR) probes, finding that they exhibit differences but generally fall into three classes. Low levels of *mew* can be detected in the CVM cells as they are specified and extend beyond stage 13, overlapping with *HLH54F* and *zfh1* expression; these three genes make up the continuously expressed contingent (Fig. 2A, shown for stages 10-13). In contrast, genes *sna*, *kon* and *Doc2* initiate expression at stage 10 or earlier, with varied dynamics (Fig. 2B). *sna* is expressed in the CVM progenitor cells (stage 5) (Ismat et al., 2010) and continues to be expressed in CVM progenitors until expression is extinguished after stage 10 (Fig. 2B). Meanwhile, *kon* is expressed at stage 8, becomes enriched in a subset of cells located at the migrating front, and then expands expression to all cells later to support muscle fusion (Fig. 2B). A third set of genes initiates expression later, around stage 11, with *beat-IIb* expressed slightly earlier and *CG5080* expressed later (towards stage 12) (Fig. 2C). In summary, three dynamic trends were identified: genes are (1) co-expressed with *HLH54F* continuously throughout the entire CVM development (‘continuous’; Fig. 2A), (2) initiated by stage 10 before the beginning of cell migration (‘early’; Fig. 2B) or (3) initiate at stage 11 when cells are actively migrating (‘late’; Fig. 2C).

To determine whether enhancer activity reflects these dynamic gene expression patterns, we compared reporter expression supported by the ten enhancers to that of the endogenous genes they normally regulate. Reporter expression driven by enhancer sequences associated with genes *HLH54F*, *sna*, *Doc2*, *beat-IIa*, *beat-IIb* and *CG5080* was found to fully recapitulate the endogenous expression of the respective genes (compare Fig. 2A-C with Fig. 1D). However, for some genes (i.e. *mew*, *zfh1* and *kon*), the identified enhancer represents only a subset of the temporal expression pattern of the respective gene. For example, the *mew* enhancer is only expressed late (Fig. 1D, see *mew.v-Gal4*), but the gene is continuously expressed (Fig. 2A, *mew*). This suggests that another enhancer, currently uncharacterized but possibly relating to other peaks occupied by TFs, likely supports the early expression pattern of *mew*.

To obtain independent confirmation of the dynamic trends identified by *in situ* hybridization, we examined a single-cell (sc) RNA-seq dataset produced in a previous study (Sun et al., 2023). Uniform Manifold Approximation and Projection (UMAP) technique in Seurat (Stuart et al., 2019) was used to reduce the dimensionality of RNA-seq data of all single-cells isolated from embryos to a 2D plot that groups cells into clusters; in particular, cluster 10 relates to the CVM (Fig. 3A). Here, we further extended the analysis of cluster 10 by conducting Monocle2 pseudotime analysis to identify multiple cell states. This approach applies machine learning to single-cell transcriptomic data to order cells by their relative progression over a trajectory, or in pseudo-developmental time (Trapnell et al., 2014; Qiu et al., 2017). UMAP visualization of pseudotime analysis suggests younger cells of the cluster are located at the base (cyan) on the UMAP plot in comparison with older cells that are located at the tip (dark blue) (Fig. 3A′). This pseudotime model, coupled with UMAP display of expression data for individual genes, suggests expression of *sna*, *kon* and *Doc2* is associated with younger cells (‘early’; pseudotime ∼10), and expression of *beat-IIb*, *beat-IIa* and *CG5080* is associated with older cells (‘late’; pseudotime ∼15) (compare Fig. 3C,D with Fig. 3A′). In contrast, *HLH54F*- and *mew-*positive cells are associated with a larger fraction of cells within this cluster, suggesting these genes are expressed continuously, including at early and late stages (compare Fig. 3B with Fig. 3A′; ‘continuous’, pseudotime ∼10-15). Likewise, *zfh1* is continuously expressed in the CVM, associated with all cells expressing *HLH54F*. *zfh1* is also broadly expressed throughout the mesoderm both within and outside cluster 10, and later on in other muscle subtypes (compare Fig. 3B with Fig. 3A′; Lai et al., 1991; Postigo and Dean, 1997). *Syn2* transcripts were not detected by the scRNA-seq. In all, the scRNA-seq provides a parallel approach to *in situ* hybridization (see Fig. 2) in identifying genes expressed in the CVM as well as to discovering differences in their timing of expression (Fig. 3A-D).

We also explored expression of other TFs within the CVM using this scRNA-seq dataset. For example, the *byn* gene has been shown to impact CVM cell migration and survival (Kusch and Reuter, 1999). However, comparisons of the *sna* and *byn* UMAP profiles (Fig. 3C,E) suggest that expression of *byn* ceases before that of *sna*, which occurs at stage 10. Although we cannot eliminate the possibility that the Byn protein product perdures, we decided to focus on genes that are highly expressed in migrating CVM cells.

### Comparative studies of enhancer sequences and identification of over-represented motifs

To provide more insight into the gene regulatory networks (GRNs) controlling gene expression dynamics in the CVM, we used computational tools to identify binding sites for TF inputs within the enhancer sequences supporting expression in CVM cells from stages 10-13. Using a computational approach involving motif discovery and enrichment analysis (XSTREME), we scanned all ten enhancer sequences for over-represented sequences of 6-15 bp in length and five over-represented motifs of high significance were found (e≤0.05; see Materials and Methods). Two motifs appear similar to the consensus binding site for HLH54F and Biniou (Fig. 3G, HLH54F-like and Biniou-like), factors that have documented roles in the CVM, including supporting expression through the *doc2* enhancer (Zaffran et al., 2001; Ismat et al., 2010); the other motifs match the consensus binding sites of Dorsal, a TF required for embryonic dorsal-ventral patterning (Reeves and Stathopoulos, 2009), and CG5953, a predicted DNA-binding protein containing an N-terminal MADF domain (Fig. 3G, Dorsal/Lola and CG5953). Dorsal acts early in the cellular blastoderm stage to support expression in ventral regions of the embryo where CVM precursor cells are specified. CG5953, on the other hand, is poorly characterized, but its consensus DNA-binding sequence was identified in a large-scale study using bacterial one-hybrid assays to survey the DNA-binding properties of a number of proteins with predicted DNA-binding domains (Shazman et al., 2014). The last sequence motif remains undefined (Fig. 3G, unknown) as it failed to match any entry in the JASPAR database of binding elements.

The CG5953-binding motif is associated with nine out of 10 enhancers (Fig. 3H), and both scRNA-seq data and *in situ* hybridization show that *CG5953* is expressed in CVM cells from stage 11 onwards throughout active migration (Fig. 3E,F). However, in mutant embryos lacking *CG5953*, only minor migration defects were observed: a small number of CVM cells veer off-track at stage 13 and expression of late gene *CG5080* appears slightly delayed (Fig. S2). Considering that other MADF TFs are expressed in the mesoderm (e.g. Mes2), it has been hypothesized that these factors may function redundantly (Zimmermann et al., 2006), which may explain why the loss of *CG5953* is not associated with a stronger CVM cell migration phenotype.

Although this enhancer analysis suggests that HLH54F, Biniou, Dorsal/Lola and CG5953 are general inputs acting to support gene expression in the CVM cells, as motifs are associated with most enhancer sequences, no particular motif was found to be associated with a specific class of enhancers (i.e. continuous, early or late).

### The influence of the cell cycle on CVM cell dynamic gene expression

**Fig. 4.**
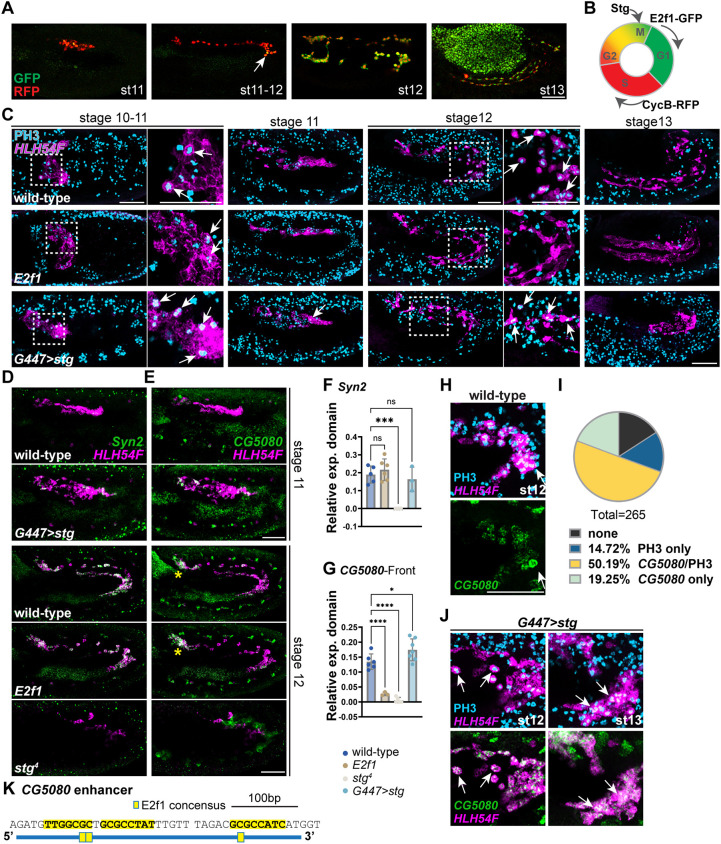
**Cell cycle progression influences expression dynamics of a subset of genes in the CVM, including *CG5080.*** (A,B) Cell cycle progression shown by the Fly-FUCCI system (Zielke et al., 2014; Macabenta et al., 2022) in embryos when *G447-GAL4* is used to drive CVM-specific reporter expression and antibodies are used to detect RFP and GFP signals. Anti-RFP indicates cells during S phase (red), anti-GFP shows cells at G1 phase (green), and merged signal (yellow) shows cells at G2 to M. (C) Anti-PH3 staining (light blue) indicates the cell division patterns in the CVM migrating cohort (stage 10-13) in wild-type and mutant embryos. Arrows indicate the dividing cells with nuclear PH3 signals, and *HLH54F* hybridization chain reaction (HCR) probe labeling CVM cells (magenta). Areas outlined are shown at higher magnification on the right. (D,E) *CG5080* and *Syn2* (green) colocalizing with *HLH54F* (magenta) by HCR *in situ* hybridization in wild-type and cell cycle-disrupted mutant embryos at stages 11-12. (F) Quantification of relative expression of *Syn2* (D, green) in *HLH54F*-positive CVM cells (D, magenta) detected by HCR probes in control and mutant embryos. (G) Quantification of relative expression of *CG5080* (E, green) in CVM cells at the front of the collective (E, magenta). Two-tailed one-way ANOVA with Dunnett's multiple comparisons test was performed (*P<0.05, ***P<0.001, ****P<0.0001). (H-J) Strong *CG5080* expression (green) correlates with cell division (PH3, light blue) that takes place at the posterior turn of stage 12 embryos [arrows in H (wild type); quantified in I (265 cells, *n*=10 embryos); see Materials and Methods] or additionally at stage 13 in embryos overexpressing *stg* [arrows in J (*G447>stg*)]. (K) E2F1 consensus binding site matches within the *CG5080* enhancer, supporting expression in CVM cells. Anterior to the left and dorsal upwards. Scale bars: 50 μm.

CVM cells proliferate during migration and cell cycle regulators have been shown to play important roles in coordinating expression of genes relating to cell proliferation, growth, apoptosis, metabolism and differentiation (Müller et al., 2001; Cam et al., 2004). As the first G_1_ phase accompanied by E2F1 function in the CVM emerges during the course of CVM migration (Macabenta et al., 2022), we hypothesized that cell cycle progression may present additional regulatory inputs responsible for the distinction of late versus early CVM gene expression. We have previously shown that a FUCCI reporter in which RFP and GFP signals are indicative of S [RFP-Cyclin B (CycB)] and M/G1 [GFP-E2F Transcription Factor 1 (E2F1)] phases, respectively, indicates cell cycle progression within CVM cells over the course of their migration (Fig. 4A,B; Zielke et al., 2014; Macabenta et al., 2022). We observed that the GFP signal becomes fully apparent as CVM cells divide at stage 12, corresponding to the period when late genes are expressed (i.e. *beat-IIa*, *Syn2* and *CG5080*).

We therefore investigated whether late genes might be influenced by the cell cycle (compare Fig. 4A with Fig. 2C). Cell division within the CVM can be detected using the anti-phosphorylated Histone 3 (PH3) antibody, an indicator for cells in M-phase (Shibata et al., 1990), coupled with an antibody to Teyra-meyrha (Tey), a nuclear protein, to label CVM cells specifically (Stepanik et al., 2016). Normally migrating CVM cells divide at two locations: at the very back when cells just migrate out (stages 10-early 11), and at the posterior turn at stage 12 (Fig. 4C, wild-type stage 12, arrows). Expression of several late genes (*Syn2* and *CG5080*) is delayed in *stg* mutants: they are absent up to stage 12 (Fig. 4D-G, *stg^4^*) and are expressed only later at stage 13 (Fig. S3F). Overexpression of *string/Cdc25* within CVM cells using the Gal4/UAS system (Caygill and Brand, 2016) leads to increased or ectopic cell division in the CVM at stage 11 and before reaching the posterior turn at stage 12 (Fig. 4C, *G447>stg*, arrow; see also Fig. S3A). Consequently, this also leads to a slight increase in *CG5080* expression at stages 11-12 (Fig. 4E,G, *G447>stg*; see Materials and Methods); in contrast, *Syn2* expression does not change (Fig. 4D,F, *G447>stg*). This initiation of *CG5080* expression in CVM cells correlates with the transitioning of the cell through M phase (marked by PH3 staining), both in wild type (Fig. 4H,I) and when *stg* is overexpressed in the CVM cells (Fig. 4J). *CG5080* appears to be more sensitive to levels of *stg* and cell cycle progression than the other late genes.

As expression of *hid* also increases around stage 12, when CVM cells divide in an E2F1-dependent manner (Macabenta et al., 2022), we hypothesized that E2F1 might act as a general positive input to support the expression of other late genes, such as *CG5080*, in addition to *hid*. Scanning the identified enhancers, we found that matches to the E2F1-binding consensus are present within the late gene enhancers but absent from those of the early genes (Fig. S3B). In *E2f1* mutants, a decrease in cell division is observed at stage 12 (Fig. 4C, *E2f1*) and, concomitantly, expression of *CG5080* is significantly diminished in the migrating CVM cells (Fig. 4E,G, *E2f1*; also Fig. S3G). Little effect is observed on *Syn2*, *beat-IIb* and *beat-IIa* expression (Fig. 4D,E and Fig. S3D,E, *E2f1*). E2f family TFs constitute one of several regulators of cell cycle-dependent transcription, each of which is controlled by specific cyclin-dependent kinases (CDKs) that regulate discrete gene expression programs (Zhang et al., 2021). Furthermore, the *CG5080* enhancer has three matches to the E2F1 consensus binding motif (Fig. 4K), which possibly explains why this gene is more dependent/sensitive to levels of this one particular factor and cell cycle state. The late genes might have different dependencies on specific TFs to modulate cell cycle-dependent expression or otherwise control their expression from stage 11 onwards (see Discussion).

### Regulation of early gene expression spatial extent by TFs Zfh1, Sna and Doc2

Although cell cycle progression/E2F1 impacts late gene expression, it is not required to support early gene expression (e.g. *Doc2*; Fig. S3C). To provide additional insight into the regulation of early gene expression in the CVM, we focused on the TFs that are expressed either continuously (*zfh1*) or early (*sna* and *Doc2*). These genes are associated with CVM cells from stages 10 to 12. In particular, we were interested in understanding how these TFs might control polarized gene expression within the migrating collective, as *zfh1* and *sna* are ubiquitously expressed, whereas *Doc2* is localized to the anterior end of the migrating collective (Figs 1B and 2B).

**Fig. 5.**
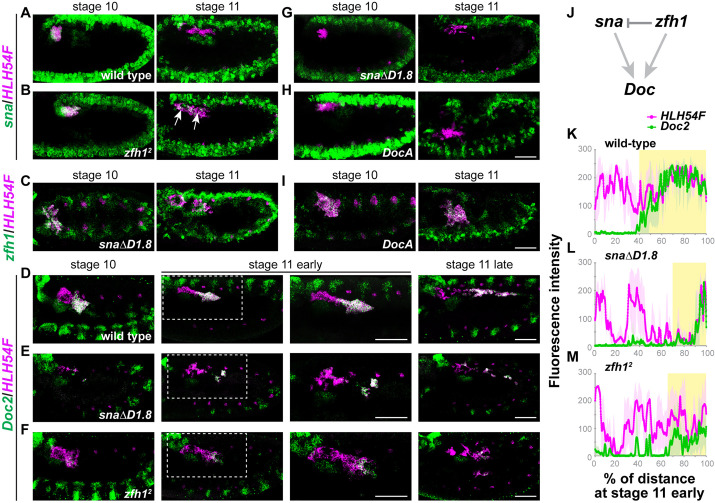
**Phenotypes associated with mutants for TFs expressed early in development: s*nail*, *zfh1*, *Doc1*, *Doc2* and *Doc3*.** (A-I) CVM cells are labeled with *HLH54F* probes in magenta; expression of *sna*, *zfh1* and *Doc2* are detected with hybridization chain reaction probes in green. (A,B,G,H) Deletion of the *sna* distal enhancer results in a loss of *sna* expression in the CVM cells (G) that is normally observed at stage 10 and restricted to the very posterior cells at stage 11 (A). Loss of *zfh1* leads to an increased *sna* expression that persists to stage 11 (arrows, B; slightly older stage than A). (C,I) *zfh1* expression is unaffected by loss of *sna* or Doc genes; see Fig. 1B for control. (D-F) Both *zfh1* and *sna* are required for maintaining the expression of *Doc2* in the migrating CVM cells. Magnified views of the outlined area are shown on the right. (J) Diagram showing the regulatory relationship between *sna*, *zfh1* and Doc genes (*Doc1*, *Doc2* and/or *Doc3*). (K-M) Quantification of *Doc2* expression in the CVM cells (D-F, stage 11 early) as measured by its fluorescence intensity profile from the anterior (0%) to posterior (100%) of the migrating collective. Profile of *HLH54F* is also shown to locate CVM cells (see Materials and Methods). *n*=3, shaded area showing error bar calculated with standard deviation. Yellow color highlights the region of co-expression of *Doc2* (green) and *HLH54F* (magenta) in the CVM cells. Anterior to the left and dorsal upwards. Scale bars: 50 μm.

To assay mutant phenotypes for *zfh1* and *Doc*, we used available mutants, while assaying of *sna* loss-of-function in the CVM required generation of a novel mutant. A null mutant is available for *zfh1*, as is a deficiency mutant, *DocA*, that removes *Doc2* as well as paralogs *Doc1* and *Doc3*; both mutants have been previously characterized (Lai et al., 1993; Reim et al., 2003). The ∼1.8 kB *snaDistal* enhancer is required to support the expression of *sna* in the Malpighian tubules/CVM (Dunipace et al., 2011). Mutants with a deletion of this *snaDistal* enhancer segment (*snaΔD1.8*) are viable and exhibit relatively normal gastrulation (Irizarry et al., 2021 preprint). Importantly, CVM cells are specified, although fewer relative to wild type, and *sna* expression is missing at stage 10/11 (Fig. 5G, compare with Fig. 5A).

Using these mutants for *zfh1*, *Doc* and *sna* (i.e. *zfh1^2^*, *DocA* and *snaΔD1.8*), we sought to determine the relationships between these factors in the context of transcriptional regulation in migrating CVM cells. *sna* expression persists longer in *zfh1* mutants as cells initiate migration but remains relatively unchanged in the *DocA* mutant background (Fig. 5A,B,H). *zfh1* expression is unaffected in either mutant (Fig. 5C,I), whereas *Doc2* expression is reduced in both *sna* and *zfh1* mutant backgrounds, albeit in different ways (Fig. 5D-F). Using a quantitative analysis to assay gene expression throughout the migrating collective (see Materials and Methods), we find that the average *Doc2* expression is enriched to the anterior ∼60% of the migrating CVM cell collective, as indicated by the overlap with *HLH54F* signals (Fig. 5D,K). In the *snaΔD1.8* mutant, *Doc2* expression is decreased, apparent only in ∼10% of cells at the front (Fig. 5E,L). Loss of *zfh1* also causes *Doc2* expression to decrease in both levels and fraction of cells: reduced *Doc2* expression is present in the approximately anterior 20% of the cells (Fig. 5F,M). These results suggest a gene regulatory program in which Zfh1 represses *sna*, and both Zfh1 and Sna act to promote the expression of *Doc2* (Fig. 5J)*.*

As Zfh1, Snail and Doc2 TFs are expressed early during CVM cell migration, we investigated whether they influence late gene expression perhaps by using a feed-forward mechanism. However, expression dynamics of late genes (i.e. *beat-IIa*, *Syn2* and *CG5080*) appear largely normal in *zfh1^2^*, *snaΔD1.8* and *DocA* mutants with transcripts present in the CVM from late stage 11 onwards, as found in the wild type (Fig. S4).

### *zfh1*, *sna* and Doc genes regulate spatially localized early gene expression within migrating CVM cell collectives

**Fig. 6.**
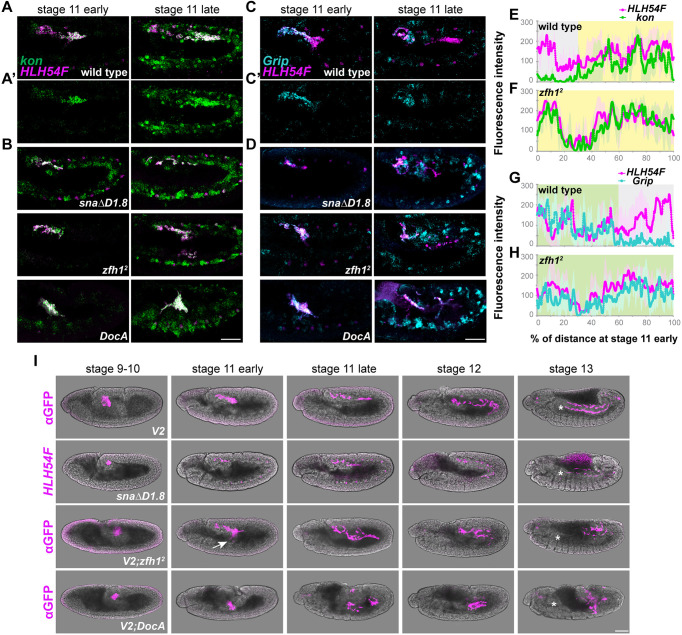
***sna*, *zfh1* and *DocA* mutants exhibit defects in polarized gene expression within the migrating CVM cell collective but have limited effects on timing of gene expression.** (A-D) *kon* (A,A′,B) and *Grip* (C,C′,D) expression in the CVM cells (labeled by *HLH54F*), as detected by hybridization chain reaction *in situ* hybridization in wild-type, *snaΔD1.8*, *zfh1^2^* and *DocA* mutant backgrounds. A′ and C′ show individual *kon* and *Grip* localizations to the front or back of the migrating collective, respectively. (E-H) Quantification of transcript levels shown as fluorescence intensity measured from posterior (0%) to anterior (100%) in the migrating collective shown in wild type versus *kon* mutant. Yellow and green shaded areas indicate overlap between *kon* and *HLH54F* (E,F) or *Grip* and *HLH54F* (G,H) signals, respectively; gray shading indicates low to no expression (see Materials and Methods). *kon* expression in all mutants is expanded (B, also quantified in F for the *zfh1^2^* mutant), whereas *Grip* enrichment in the posterior domain is only affected in *zfh1^2^* and *DocA* mutants (D,H); see also Fig. S5. (I) CVM migration assayed using anti-GFP antibody staining in wild-type, *zfh1^2^* and *DocA* mutant backgrounds carrying the *V2* transgene (*HLH54F.v2-venus*) or with *HLH54F* riboprobe and *in situ* hybridization in *snaΔD1.8* mutant. Arrow indicates specific CVM cells in the *zfh1^2^* mutant that mismigrate, moving ventrally instead of anteriorly, whereas CVM migration in *DocA* mutants is severely and generally disrupted. At stage 13, asterisks indicate the boundary between the first and second thoracic segments (i.e. T1/T2 boundary), which is the normal anterior-most position that CVM cells migrate to (Urbano et al., 2011). Anterior to the left and dorsal upwards. Scale bars: 50 μm.

In a previous study, we found that genes *kon* and *Grip* are expressed at the front and back of the migrating collective, respectively (Fig. 6A,A′,C,C′; Sun et al., 2023). Using our quantitative method for analysis of polarized expression (see Materials and Methods), *kon* transcript is detected at the anterior of the migrating collective in a domain representing ∼60% of cells at early stage 11 (yellow, Fig. 6E), similar to the domain encompassed by *Doc2* (Fig. 5K). In contrast, *Grip* transcript is detected at the posterior in a domain representing ∼60% of cells located at the back of the migrating collective (green shaded area, Fig. 6G) and becomes further restricted to the posteriormost ∼40% of cells by late stage 11 (green shaded area, Fig. S6D). Although the HCR probes used are incompatible for detecting colocalization, these results suggest the expression of *kon* and *Grip* gradually become non-overlapping during stage 11.

As *Doc2* transcripts specifically are localized to the anterior (not *sna* or *zfh1*), we hypothesized that *Doc2* regulates the localized expression of other early genes, i.e. *kon* and *Grip*. In wild-type embryos, *Grip* and *kon* are expressed early in distinct domains, as the CVM cells initiate migration until late stage 11 before cells divide (Fig. 6A,A′,C,C′; Sun et al., 2023). In *DocA* mutants, we found that *kon* and *Grip* are no longer differentially localized but are instead broadly expressed in all CVM cells (Fig. 6B,D, *DocA*; Fig. S5C,D), supporting the view that Doc2 acts upstream of *kon* and *Grip*, and is necessary to promote their spatially localized expression patterns.

As *snaΔD1.8* and *zfh1* mutants exhibit decreased *Doc2* expression (Fig. 5E,F,L,M), we expected to see effects on localization of *kon* and *Grip* similar to those in *DocA* mutants. Both *snaΔD1.8* and *zfh1^2^* mutants fail to localize *kon* to the front, with transcripts instead detected in all CVM cells (Fig. 6B, *snaΔD1.8* and *zfh1^2^*; Fig. 6E and Fig. S5A). Surprisingly, different effects were observed for *Grip*. Although *Grip* expression in the *zfh1^2^* mutant is uniform (Fig. 6H *zfh1^2^*), its localization at the back in the *snaΔD1.8* mutant is similar to that in the wild type (Fig. S5B). Collectively, these results suggest that Doc, Sna and Zfh1 contribute to anterior localization of *kon*, but that only Doc and Zfh1 (not Sna) are required to support localization of *Grip* to the back.

We postulate that the level of polarity retained within the migrating collective may relate to the range of cell migration defects associated with the mutants examined (Fig. 6I). CVM cells in the *DocA* mutants exhibit no polarity (i.e. overlapping *kon* and *Grip*) and are bunched together. As the ectoderm patterning is also affected, their phenotype is difficult to interpret. Comparing *zfh1^2^* to *snaΔD1.8* mutants, we found that CVM cells in *zfh1^2^* mutants completely lose their polarity (i.e. *Grip* also failed to localize to the back), veer off-track, divide ectopically and never reach the anterior midgut (Fig. 6I, arrow; *zfh1^2^*). Therefore, Zfh1 may play a more important role in regulating the invasive behavior of CVM cells compared with *sna* (see Discussion).

### Transition from early to late gene expression is facilitated by cell division

**Fig. 7.**
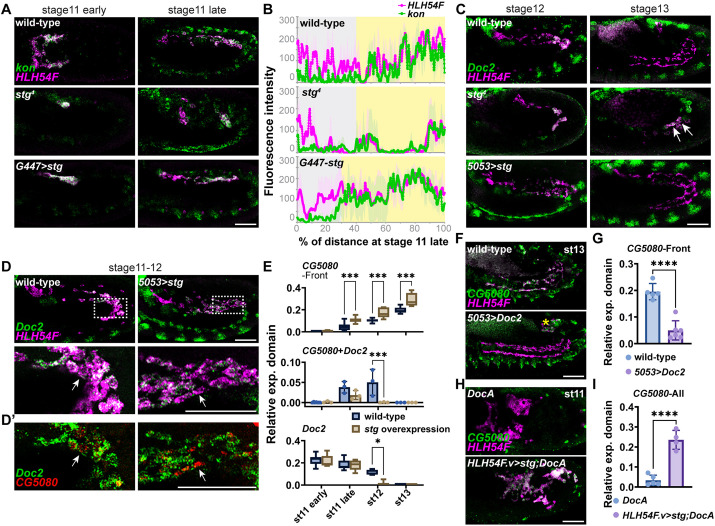
**Disrupting cell cycle progression has little effect on polarized gene expression but cell cycle progression and loss of Doc genes accelerate *CG5080* expression.** (A,C) Expression of *kon* (green, A) or *Doc2* (green, C) in CVM cells (*HLH54F*, magenta) detected by *in situ* hybridization with hybridization chain reaction (HCR) probes in wild-type and cell cycle mutants: *stg* mutant (lacks cell division) or ectopic expression of *stg* (i.e. *G447>stg*; supporting extra cell division). (B) Quantification of *kon* localization in the CVM (stage 11 late, shown in A) as measured by its fluorescence intensity profile from anterior (0%) to posterior (100%) along with *HLH54F* (see Materials and Methods). Yellow shaded areas indicate overlap between *kon* and *HLH54F*, whereas gray shading indicates low to no expression. (D) HCR *in situ* hybridization showing overexpression of *stg* (*5053>stg*) speeds up the transition from *Doc2* (green) to *CG5080* (red, D′) expression in CVM cells (magenta). The outlined areas are shown at higher magnification in the lower panels. Arrows indicate cells in which *Doc2* expression is low but *CG5080* is high. (E) Quantification of D indicates that *Doc2* and *CG5080* show opposite trends over time; the switch is earlier upon ectopic expression of *stg* (*5053>stg*). Two-tailed two-way ANOVA test was performed with corrections for multiple comparisons using statistical hypothesis testing. For CG5080-Front, *n*=8, 8, 8 and 6 (at stage 11 early, stage 11 late, stage 12 and stage 13, respectively, in wild type); *n*=6, 8, 6 and 5 (in *5053>stg*). For *Doc2*, *n*=8, 8, 5 and 6 (in wild type); *n*=6, 6, 5 and 5 (in *5053>stg*). *P<0.05; ***P<0.001. Individual data points are provided when *n*<5. (F,G,) *CG5080* levels are decreased relative to wild type when continuing expression of *Doc2* in the CVM is forced by *5053-GAL4* (F) and quantification measuring the difference (two-tailed unpaired *t*-test, *P*<0.0001, G). (H,I) In the *DocA* mutant, *CG5080* is not expressed at stage 11; however, when *stg* is ectopically expressed in the CVM of *DocA* mutants using a *HLH54F.v-GAL4* driver, then *CG5080* expression is initiated early throughout the CVM (two-tailed unpaired *t*-test, ****P<0.0001, I). Anterior to the left and dorsal upwards. Scale bars: 50 μm.

As we had identified cell division as a pivotal regulator of late gene expression, we next investigated whether precocious cell division affects spatially localized expression of the early genes within the migrating collective. To provide insight, we examined *kon, Grip* and *Doc2* expression in *stg* mutants (*stg^4^*) or upon *stg* ectopic expression (G447>*stg*) at late stage 11. Assaying gene expression polarization in the migrating collective slightly later will ensure that the drivers of ectopic expression (*G447-GAL4*) are given adequate time to induce cell division. In *stg* mutants, despite the block to the cell cycle, *kon* remains localized to the front and *Grip* to the back, suggesting that proper spatially localized gene expression is maintained; a similar result is observed upon *stg* ectopic expression (*G447>stg*) (Fig. 7A,B and Fig. S6). Therefore, modulating when cell division occurs during CVM migration does not affect the polarized expression of early genes, and polarity is retained even when fewer cells are present in the migrating collective.

Alternatively, as *Doc2* expression is normally lost by stage 12, we hypothesized that the cell cycle might influence when early genes are extinguished by shutting off *Doc2*. We assayed *Doc2* expression in *stg* mutants or upon *stg* overexpression (*5053>stg*), noting that *5053-GAL4* and *G447-GAL4* drivers are roughly equivalent, whereas *HLH54F.v-GAL4* acts both earlier and stronger in terms of driving expression in CVM cells (see Materials and Methods, also Fig. 1D). Indeed, we found that *Doc2* remains expressed for longer, extending into stage 13, in *stg* mutants (Fig. 7C, compare *stg* with wild type). Furthermore, levels of *Doc2* appear lower upon *stg* overexpression compared with wild-type embryos at stage 12 (Fig. 7C,E, *5053>stg*). These results support the view that cell division is likely one of several cues that facilitate the transition of CVM gene expression programs from one state to the next (i.e. early to late gene program) and may act by regulating Doc gene expression.

To determine whether the early and late gene expression programs are mutually exclusive and coordinated by cell division, we selected one gene from each program that appears to be sensitive to the manipulation of the cell cycle: *Doc2* (early) and *CG5080* (late). First we investigated whether these genes are ever co-expressed in the CVM cell during the transitioning period, finding co-expression is limited to stage 11 late and stage 12 (Fig. 7E, *CG5080-Doc2*). *Doc2* trends down with time, as *CG5080* increases. These opposite trends support the idea that these two programs (i.e. early versus late gene expression in the CVM) are coupled. Furthermore, when we introduce ectopic cell division in the CVM, the transition between loss of *Doc2* and gain of *CG5080* occurs earlier (Fig. 7E). Upon overexpression of *stg*, the early gene *Doc2* is downregulated in the CVM cells and is extinguished before stage 12 (Fig. 7D,E, *Doc2*), and the cells that lose *Doc2* are positive for *CG5080* (Fig. 7D,D′, arrows indicate cells that express *CG5080* but not *Doc2*).

Doc2 appears to repress the late gene expression program. When continuous *Doc2* expression is forced using *5053-GAL4*, *CG5080* is repressed with minimal levels detected in the CVM (Fig. 7F,G). However, loss of *Doc* function alone does not lead to a precocious activation of the late genes, suggesting loss of this repressor is not enough to support earlier gene activation (Fig. S4, *DocA*). We also found that although overexpression of *stg* leads to increased expression of *CG5080* it cannot support earlier expression (Fig. 7E, *CG5080*). We hypothesized that two requirements must be met to promote late gene expression, as elimination of Doc2-mediated repression is permissive whereas cell division is the accelerator. In either wild-type or *DocA* mutant background, robust *CG5080* is not detected until stage 12 (Fig. S4 and Fig. 7E). However, when *stg* is overexpressed in the *DocA* mutant background using *HLH54F.v-GAL4*, strong *CG5080* expression is observed in CVM cells at early stage 11 (Fig. 7H,I). Therefore, both cell cycle progression and loss of *Doc2* expression are required to activate the late gene expression, and our results support the view that two mutually exclusive gene expression programs connected by cell division act sequentially to control the behavior of CVM cells during migration.

## DISCUSSION

Unlike other forms of collective migration that rely on relatively stable contact and adhesion between neighboring cells, during the first half of their migration CVM cells have more transient dynamic interactions with their neighbors, likening the process to cell streaming; this changes during the second phase of migration subsequent to cell division, when CVM cells form more stable intercellular interactions with each other and with the substrate, all the while continuing forward migration. Collectively, our results demonstrate that the early-expressing TFs Zfh1, Sna and Doc play crucial roles in regulating spatially localized gene expression in actively migrating CVM cells, and the polarized expression of these factors might promote directed cell migration. In particular, we suggest that Zfh1 acts to regulate an invasive gene expression program during the first half of CVM cell migration. Zfh1 acts as a repressor, and in *zfh1* mutant embryos CVM cells appear to be highly protrusive and branched (Fig. 6I, *zfh1*). Conversely, in addition to supporting spatially localized expression in CVM cells at an early timepoint, *Doc2* acts to repress late genes such as *CG5080*. As the early genes control the migratory behaviors of the CVM cells, we suggest that late genes such as *CG5080*, an uncharacterized gene with homology to vertebrate laminin β subunit 1 (LAMB1), might be involved in the determination of visceral muscle cell fate. It is possible that the programs driving early vs late gene expression are mutually exclusive and perform different activities that can be explored in future studies.

**Fig. 8.**
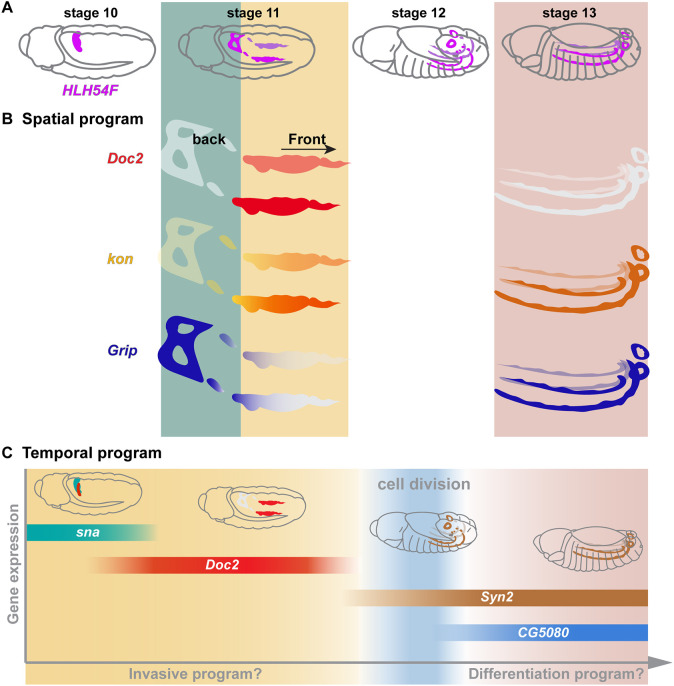
**Schematic summarizing spatial and temporal gene expression programs in migratory CVM cells demarcated by cell division and possible additional factors.** (A) Schematic showing the course of HLH54F-expressing CVM cell migration. (B) Spatial program schematics illustrating polarized localization of CVM genes before cell division (stage 11) and uniform distribution after cell division (stage 13). (C) Schematic illustrating dynamic and temporally restricted expression of genes before and after cell division, which normally takes place at stage 12. Temporally restricted *CG5080* expression is established by a combination of positive regulation by cell division and repression by Doc2*.*

In addition, our data suggest cell division acts to delineate the early spatially localized gene expression program from the late one (Fig. 8A), allowing a reset of the transcription state to facilitate changes in cell behavior, e.g. cell attachment or invasion (Fig. 8B). Such a role for cell division has been identified for other migrating cell types; for example, in *C. elegans*, anchor cell division is associated with a change in epigenetic cell state (Matus et al., 2015). Chicken cranial neural crest cells maintain mitotic quiescence during the early phase of their migration until invading the branchial arches, where they differentiate into a variety of cell types (Ridenour et al., 2014).

Our finding that E2f-binding motifs are represented in many of the late CVM enhancers identified is consistent with previously published research describing how the E2F family of transcriptional regulators can also be repurposed for roles outside a cell cycle context in post-mitotic cells, including controlling myogenic differentiation (Zappia and Frolov, 2016). In adult *Drosophila* skeletal muscle cells, knockdown of the E2F co-factor DP TF (Dp) did not result in defects in cell proliferation and myoblast fusion, but presented defects in muscle growth; indeed, the expression of structural genes, including *Myosin heavy chain* (*Mhc*) and *Tropomyosin 1* (*Tm1*), and regulators such as *held out wings* (*how*) and *Mef2* was found to be reduced in Dp-depleted skeletal muscle tissues (Zappia and Frolov, 2016). Most notably, cell cycle regulators such as E2F1, Dp and Retinoblastoma family protein (Rbf) were found to be highly enriched in promoters for the myogenic loci, even before the activation of the genes themselves, suggesting that these regulators are necessary for expression of these genes, but are not sufficient for activation. Our study adds to the growing body of evidence that TFs such as E2F1, which are most well-known for their role in cell cycle regulation, can also cooperate with other tissue-specific TFs to control gene expression, including those required for terminal differentiation. There is an intriguing possibility that this is a conserved mode of gene regulation, as mammalian E2f3 has been shown to directly regulate myogenic differentiation outside an apparent cell cycle function (Asp et al., 2009).

Balancing proliferation and motility is key to cell migration, and how proliferation contributes to the process depends on the cell type as well as the mode of migration, i.e. single cell versus collective. Although it could be crucial for maintaining the primordium size and generating polarity or cellular diversity within the migrating collective, cell division and migratory/invasive behavior are at times incompatible. For example, differentiation follows an invasive cell state and takes place only after mitosis in the worm anchor cell; highly invasive metastatic cancer cells tend to be less proliferative (Hoek et al., 2008; Gil-Henn et al., 2013; Matus et al., 2015). At least in part, this incompatibility can be explained by the distinctive cytoskeletal organization required by these two processes. Migrating cells are protrusive, with polarized branched F-actin driven by Rac and Cdc42 activities, while dividing cells become rounded during mitosis with symmetric distribution of F-actin (Dogterom and Koenderink, 2019). Furthermore, cell division, in particular G1/S progression, has been linked to cell fate decisions and can facilitate transitions between cellular states associated with different transcription signatures (Soufi and Dalton, 2016). A recent study of melanoma cells showed that an intermediate state, associated with a stable GRN enriched for epithelial-to-mesenchymal transition (EMT) genes, exists in addition to two main switchable transcriptional states/cell behaviors: a melanocytic differentiated state (MEL) and mesenchymal invasive-like state (MES) (Wouters et al., 2020). Interestingly, when the switching of these cells from MEL to MES is induced in culture, the disruption of cell cycle progression (G1 arrest) precedes activation of gene programs involved in cell migration or EMT.

Our study has identified two transcription programs, and a requirement for cell division in turning off the early program and activating the late program. The transition point from a migratory/invasive program to a differentiation/attachment program is not simply defined by shutting off one single early expressing gene; instead, multiple factors, including cell cycle regulators, are involved in progressing this transcriptional state change. During normal development, the two programs might be irreversible such that once CVM cells divide they are unable to go back to an invasive state. Understanding how cell migration programs coordinate with cell cycle progression, support invasive cell behavior, as well as ultimately promote cell differentiation has broad implications in both biology and medicine.

## MATERIALS AND METHODS

### Fly stocks and husbandry

All stocks were kept at 22°C in standard medium. Experimental crosses were kept in cages with apple juice agar plates supplemented with yeast paste. The following stocks are obtained from the Bloomington *Drosophila* Stock Center (BDSC): Fly-FUCCI (BDSC55110, *w^1118^;P{w[+mC]=UASp-GFP.E2f1.1-230}26 P{w[+mC]=UASp-mRFP1.NLS.CycB.1-266}4/CyO, P{ry[+t7.2]=en^1^}wg^[en11]^; MKRS/TM6B,Tb^1^*), *E2f1* [BDSC7274 *E2f1^i2^/TM3*,*Sb^1^* crossed into BDSC3013 *Df(3R)e-BS2,rsd^1^/TM3*,Sb^1^], *stg* [BDSC2500 *ru^1^hry^1^Diap1th^-1^st^1^cu^1^sr^1^e^s^stg^4^ca^1^/TM3,Sb^1^Ser^1^* or BDSC44368 y^1^w*;Mi{MIC}stg^MI08204^/TM3,*Sb^1^Ser^1^* crossed into BDSC25004 *w^1118^;Df(3R)BSC500/TM6C,Sb^1^cu^1^*], *zfh1* (BDSC90314 *zfh1^2^/TM3*,*Sb^1^Ser^1^*), *DocA* (BDSC91634 *Df(3L)DocA,ru^1^P{EP}smg^EP3556^/TM3*,*P{eve-lacZ8.0}AG1,Sb^1^*), *CG5953* [BDSC13223 *y^1^w^67c23^;P{lacW}CG5953^k16215^/CyO* crossed into BDSC7833 *w^1118^;Df(2L)Exel7066/CyO*], *UAS-stg* (BDSC4778 *w^1118^;P{UAS-stg.N}4*) and 5053-GAL4 (BDSC2702 w*;P{GawB}tey^5053A^/TM6B,Tb+). *HLH54F-Gap-Venus* (*V2*) (Stepanik et al., 2016), *G447-GAL4* (Georgias et al., 1997) and *snaΔD1.8* (Irizarry et al., 2021 preprint) have been previously described. *yw* is used to show the wild-type expression pattern; control refers to heterozygous *E2f1* mutant embryos from the same collections.

### Cloning of reporter constructs and generation of transgenic fly lines

Predictions for CVM-specific enhancer elements were generated using 6-8 h ChIP-ChIP data for Zfh1 (Wu et al., 2019), Bin (Zinzen et al., 2009), Mef2 (Zinzen et al., 2009) and Doc2 (Junion et al., 2012). Priority was given to regions that showed occupancy by both Bin and Mef2 while excluding Twi (Zinzen et al., 2009). Primers were generated to amplify candidate enhancers via PCR using CloneAmp HiFi PCR Premix (TaKaRa Bio) and enhancer reporter constructs were assembled by cloning amplified fragments via the pENTR/D-TOPO Gateway cloning system (Life Technologies) into pBPGUw with a *Drosophila* synthetic core promoter and *Gal4* reporter (Addgene 17575; Pfeiffer et al., 2008) using LR Clonase II (Life Technologies) or In-Fusion cloning (TaKaRa Bio) into an evep.LacZ plasmid with a heterologous minimal promoter from the *even-skipped* gene and *lacZ* reporter (Liberman and Stathopoulos, 2009). As both reporter vectors contain attB, all transgenic flies were generated using site-directed transgenesis using the line M{vas-int.Dm}ZH-2A, M{3xP3-RFP.attP′}ZH-51C (BDSC24482) by Rainbow Transgenics (Camarillo, CA, USA).

### Fixation, *in situ* hybridization and immunostaining

Embryos were fixed in 4% formaldehyde/PBST following the standard protocol for *in situ* hybridization. Riboprobes for *GAL4* and *HLH54F* were made by amplifying a ∼1.5 kb and 400 bp region from a genomic DNA sample extracted from *G447-GAL4* flies, with the reverse primers containing a T7 promoter sequence to facilitate reverse transcription with T7 polymerase (Roche, 13644022). Primers used for amplification are: GAL4_forward, ccagggatgctcttcatggattt; GAL4_reverse, ctccggaagagtagggtattg; HLH54F_forward, gagttccaccagagtagccg; HLH54F_reverse, aggcttgcacatacggaaac. The antisense RNA probes labeled with digoxiygenin (Roche, 57127420) and Biotin (Roche, 55612420) were used in combination with primary antibodies (1:400) of different origin (sheep anti-digoxigenin polyclonal antibody; Thermo Fisher, PA1-85378; RRID: AB_930545 and mouse anti-biotin, Thermo Fisher, 03-3700; RRID: AB_2532265) to detect the *in vivo* expression of target genes. Alexa Fluor (555 and 647, respectively) secondary antibodies (1:500; Molecular Probes, A-21436 and A-31571; RRID: AB_162542) were used for fluorescent signal amplification and detection.

A standard protocol was also used for antibody staining of embryos. Dilutions for primary antibodies used were as follows: chicken anti-GFP (1:1000; this work, validated by the presence of CVM-specific GFP signal in embryos carrying the V2 transgene), rabbit anti-RFP (1:400, MBL International, PM005; RRID: AB_591279), rabbit anti-Tey (1:400; Macabenta and Stathopoulos, 2019), mouse anti-PH3 (1:800; EMD Millipore, 06-570; RRID:AB_310177) and rabbit anti-Zfh1 (1:1000, a gift from Ruth Lehmann, MIT, Cambridge MA, USA). A dilution factor of 1:500 was used for the following secondary antibodies: Alexa Fluor 555 goat anti-chicken (Thermo Fisher, A-21437; RRID:AB_2535858), 488 donkey anti-rabbit (Molecular Probes, A-31573; RRID: AB_2536183) and 647 donkey anti-mouse (Molecular Probes, A-31571; RRID: AB_162542).

### Hybridization chain reaction RNA-fluorescence *in situ* hybridization

Hybridization chain reaction (HCR) RNA-fluorescence in situ hybridization (FISH) (Choi et al., 2018) was performed as described previously (Slaidina et al., 2021) with modifications to use Xylene for tissue clearing instead of 0.1% Triton X-100. HCR probes used for genes *eGFP* (order number/lot number: d21260aa-84e9-4ae2-9adf-109eef0e85eb/PRH935), *lacZ* (d5cdac09-6920-42eb-b229-b5d2421b995c/RTB830), *zfh1* (e6769188-c0db-4581-a929-39afc204d0d0/PRR866), *sna* (4614/E928), *HLH54F* (4358/E576), *Doc2* (5c204473-08ca-4546-bf24-1925b8003083/PRO669), *kon* (4049/E192), *CG5080* (5c204473-08ca-4546-bf24-1925b8003083/PRO668), *beat-IIa* (5c204473-08ca-4546-bf24-1925b8003083/PRO670), *beat-IIb* (5c204473-08ca-4546-bf24-1925b8003083/PRO671), *Syn2* (5c204473-08ca-4546-bf24-1925b8003083/PRO667), *Grip* (9d28cc4d-0e82-4330-9564-96b40a58248c/RTB563) and *CG5953* (5fa8f306-142e-4909-8555-3a57ae81b8a5/RTF900) were designed and synthesized by Molecular Instruments.

### Enhancer sequence analyses

*De novo* motif discovery was performed using XSTREME (Grant and Bailey, 2021 preprint). Enhancer sequences were manually inputted in bulk, with combined *Drosophila* databases of known motifs for analysis and discovery. Motif search was limited to results with *E*-value ≤0.05, with minimum and maximum widths of 6 bp and 15 bp, respectively. Given the set of inputted primary sequences, the *E-*value is defined as the probability of a motif being found that would discriminate the primary sequences from the XSTREME-generated control sequences at least as well, assuming a background order (*m*) value of 2 (default for XSTREME). Motifs were centrally aligned for site positional diagrams, with STREME and FIMO instructed to parse genomic coordinates; under MEME settings, the predicted occurrence for each motif was set to zero or one occurrence per sequence. Block diagrams were generated using MAST (Bailey and Gribskov, 1998) by manually inputting *de novo* motifs (see Table S1) and the 10 enhancer sequences (deposited in GenBank under accession numbers OR364904-OR364913). *E*-value threshold was set to less than or equal to 10; this is defined as the expected number of sequences in a random sequence file of the same size that would match the motif as well as the sequence does. E2f1 consensus binding sites were manually identified using published E2f1-binding motifs TTGGCGCGCATTTT (*Drosophila* biphasic motif) and TTTGGCGC (*Drosophila* E2f motif) (Georlette et al., 2007).

### Pseudotime analysis of scRNA-seq dataset

The single-cell gene expression dataset was generated in the context of a previous study (Sun et al., 2023). Pseudotime trajectory analysis for cluster 10 cells was performed using Monocle2 (Trapnell et al., 2014; Qiu et al., 2017). Pseudotime values of individual cells were projected onto a UMAP plot for visualization.

### Quantification of *Syn2*, *CG5080* and *Doc2* expression within the CVM

To assess the changes of gene expression in the CVM, we used the Squassh (segmentation and quantification of subcellular shapes) plug-in (Rizk et al., 2014) in Fiji (Schindelin et al., 2012) to quantify the fluorescent signal colocalization. Briefly, a *z*-stack of approximately 150 μm (10 slices) with the region of interest (ROI) focused on either the entire CVM population or the migrating CVM cells at the front were subjected to Squassh segmentation tool under the MOSAIC suite. Segmentation parameters were set as follows: standard deviation for *xy* 0.33 and *z* 0.79; minimum object intensity to identify CVM cells based on *HLH54F* expression 0.1; minimum object intensity for *CG5080*, *Syn2* and *Doc2* expression 0.075. Cell masking threshold for *HLH54F* was set to 0.15; for *CG5080*, *Syn2* and *Doc2*, it was set to 0.1. Size of expression domains for each gene within the ROI was calculated. Overlapping expression regions of two genes (*CG5080*/*HLH54F*, *Syn2*/*HLH54F*, *Doc2*/*HLH54*F and *Doc2*/*CG5080*) above their thresholds were also identified. The relative size of co-expression domains was calculated as percentage of the *HLH54F-* or *CG5080*-positive regions within the ROI. Statistical analysis was performed in GraphPad Prism using analysis of variance (ANOVA) for comparisons between multiple groups (genotypes or stages) and unpaired *t*-test for comparisons between two groups.

### Quantification of PH3 and CG5080 expressing CVM cells

To demonstrate cell division correlating with the expression of *CG5080*, projections of *z*-stacks from 10 wild-type stage 12 embryos co-stained for *HLH54F* and *CG5080* HCR probes, and using an antibody against mitotic marker PH3 were used for quantification. Regions containing CVM cells at the posterior turn were selected as the ROI. The number of *CG5080* and *HLH54F* double-positive, *PH3* and *HLH54F* double-positive as well as *CG5080*, *PH3* and *HLH54F* triple-positive cells were counted and calculated as ratios relative to *HLH54F*-positive cells within the ROI.

### Quantification of spatial expression domains in the CVM

To more systematically assay changes in gene expression localization throughout the migrating collectives, we devised a quantitative approach by recording the fluorescence intensity along a straight line drawn to represent the long axis of a single migrating collective – here assayed at stage 11 early for the gene *Doc2* simultaneously with *HLH54F*. Confocal fluorescent images were taken as *z*-stacks using a Zeiss LSM 800 and output as orthogonal projections. Fluorescence intensity measurement was carried out with Fuji with the ‘Multichannel Plot Profile’ function from BAR (Broad Applicable Routines) plug-in (https://zenodo.org/records/28838). A straight line was drawn along the long axis of the CVM cells to cover as many as target cells while also avoiding signals in surrounding tissues. For each genotype at a specific stage, measurements from three embryos were taken and data was output in a table format as *x*-axis represents the percentage of distance from the migration start point and *y*-axis records the relative fluorescent signal intensity. Each table was then subjected to a Matlab script for interpolation (deposited under Stathopoulos lab GitHub account, https://github.com/StathopoulosLab/intensity_interp_stats) so that an average of fluorescent intensity (*y*) at any given *x* value (0-100%) can be calculated for each genotype. Error bar represents standard deviation. Plots were generated using Microsoft Excel.

## Supplementary Material



10.1242/develop.202262_sup1 Supplementary information

Table S1 MEME motifs from analysis of 10 enhancer sequences using XSTREME. Positional weight matrices for *de novo* motifs relating to Figure 3G.
