# Supplementary material for: Two sequential gene expression programs bridged by cell division support long-distance collective cell migration
Source: Development. 2024 May 17;151(10):dev202262. doi: 10.1242/dev.202262 (PMC11165717; doi:10.1242/dev.202262)
Supplement: Supplementary information [file develop-151-202262-s1.pdf]

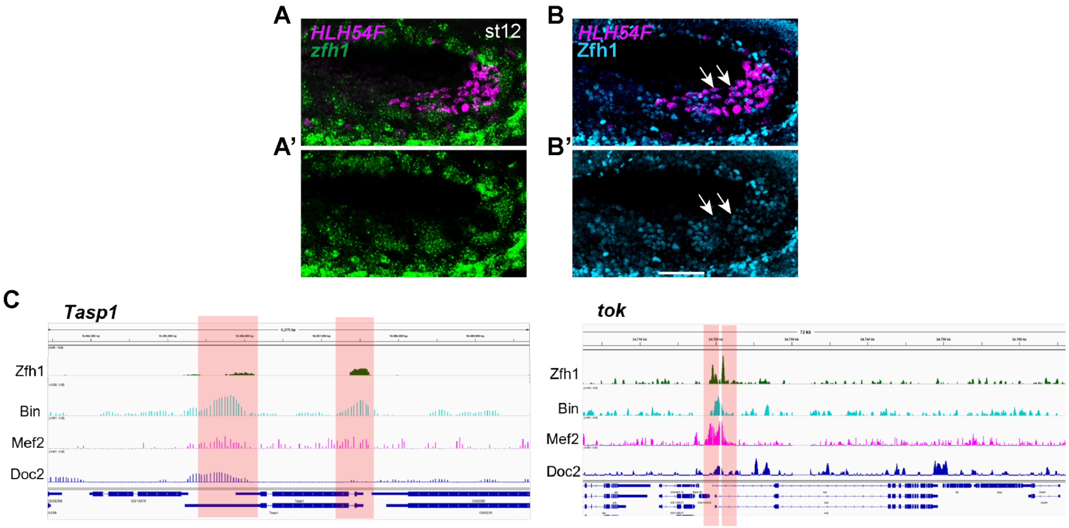

Fig. S1. Expression of *zfh1* in the CVM cells and loci containing occupied regions that fail to drive reporter expression. (A,A',B,B') Visualizing expression of *zfh1* in the CVM cells (*HLH54F*, magenta) at stage 12 by HCR in situ hybridization using a probe to *zfh1* (green) and immunohistochemistry using anti-Zfh1 antibody (cyan). Detection of *zfh1* mRNA and protein alone shown in the bottom panels (A',B'). Arrows indicate the nuclear anti-Zfh1 signals detected in CVM cells. Scale bar: 50µm. **(C)** ChIP data for TFs Zfh1, Bin, Mef2, and Doc2 at *Tasp1* and *tok* loci. Four regions assayed for enhancer activity highlighted red that all fail to drive reporter expression.

Development • Supplementary information

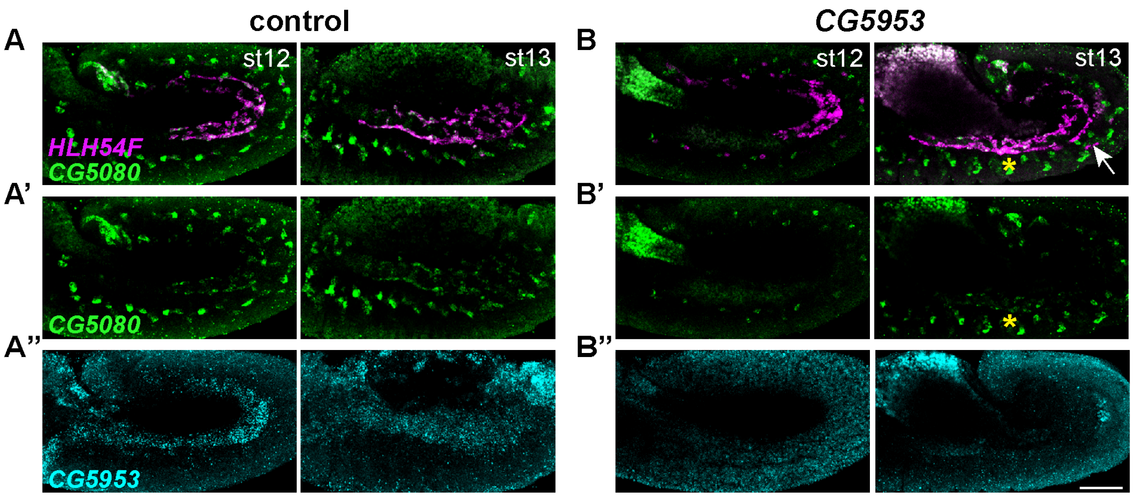

Fig. S2. Delayed expression of *CG5080* in *CG5953* mutants. *CG5080* expression (HCR probe, green) in the CVM cells (*HLH54F*, magenta) in stage 12 and stage 13 control (A,A') and *CG5953* mutants (B,B') embryos obtained from the cross of *CG5953*^{k16215}*/CyO* to *Df(2L)Exel7066/CyO*. Mutant embryos exhibit *CG5080* expression (green) later, initiating at stage 13 (asterisks, B,B') instead of stage 12 as observed in control (A,A')). While CG5953 transcripts are detected in CVM cells and possibly also in the trunk visceral mesoderm (TVM) in the control, they are absent from the mutants, shown in the bottom panels (B" compared to A"). Arrow indicates mis-migrated CVM cells. Scale bar: 50µm.

Development • Supplementary information

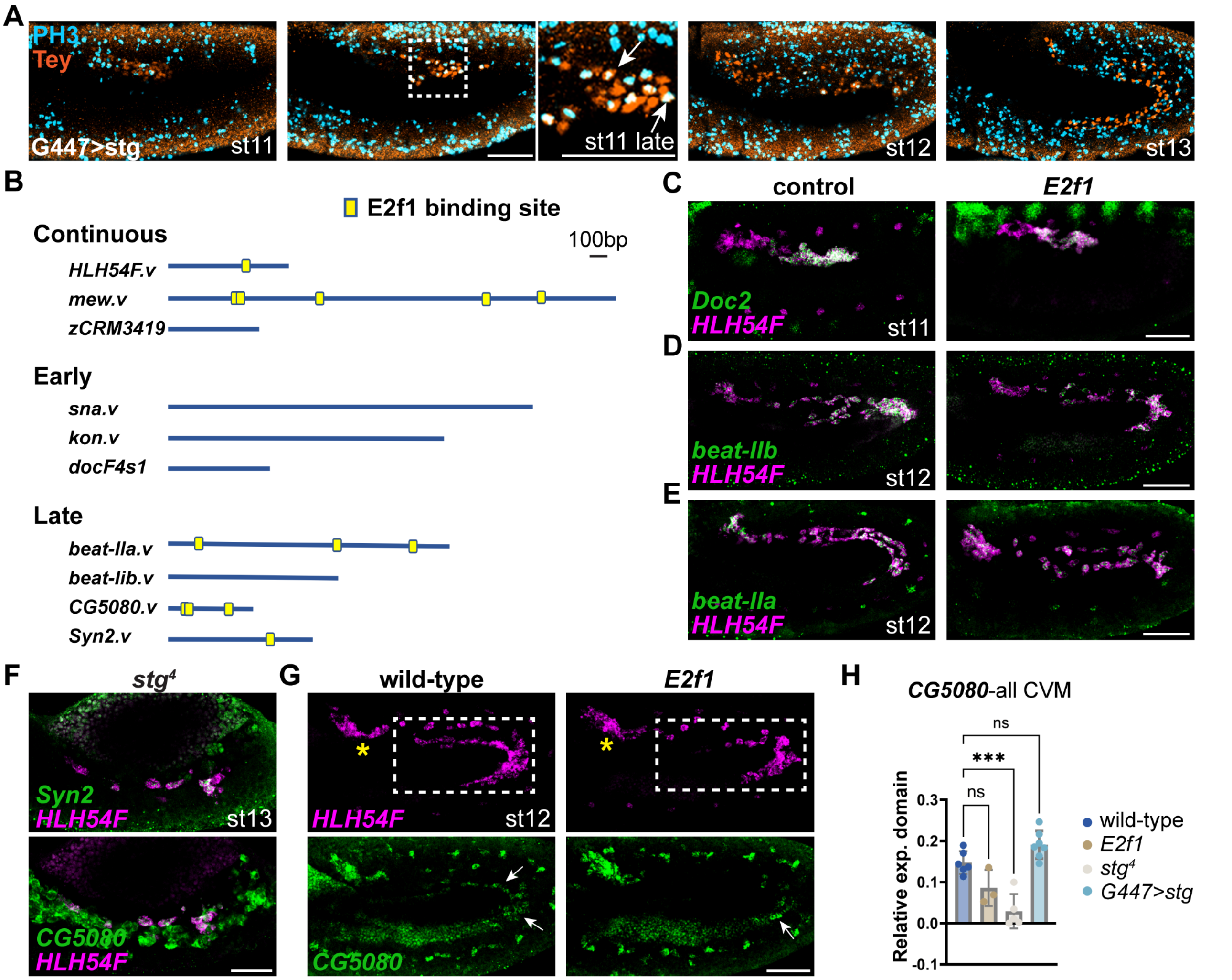

Fig. S3. The spatiotemporal dynamics of other genes expressed in the CVM are largely unaffected by loss of *E2f1*. (A) Anti-PH3 staining (light blue) indicates the cell division patterns in the CVM migrating cohort (anti-Tey positive, orange) at stage 11-13 in *G447>stg* cell division mutant embryos. Arrows point to the ectopic dividing cells with nuclear PH3 signal at stage 11. **(B)** Schematic showing identification of E2f1 consensus binding sites in CVM enhancers using published motifs TTGGCGCGCATTTT and TTTGGCGC (Georlette *et al.*, 2007)**. (C-E)**

Development • Supplementary information

Expression of CVM-specific genes are visualized by HCR in situ hybridization with *HLH54F* in magenta and other genes in green. The expression timing and patterns of early gene *Doc2* (C), late gene *beat-IIb* (D) and *beat-IIa* (E) are largely unchanged in *E2f1* mutants compared to wild-type. Control refers to the heterozygous *E2f1* mutant. **(F)** Expression of *Syn2* and *CG5080* (green) in CVM cells (magenta) is detected in *stg4* mutants by stage 13 but is absent earlier (see Fig. 4D,E). **(G,H)** Separate channel showing expression of *CG5080* (green) and *HLH54F* (magenta) detected by HCR in wild-type and *E2f1* mutants at stage 12; same embryos as shown in Figure 4D,E with channels split to show individual gene expression. Yellow asterisks indicate the back of the CVM in which *CG5080* is not dependent on E2f1 function (note the strong CG50580 signal in the mutant). Boxed regions (ROI) contain front migrating CVM cells that exhibit significantly reduced *CG5080* expression in *E2f1* mutants (arrows in the bottom panels point to CVM cells expressing CG5080, also see Methods and main Fig. 4G). When comparing CVM-specific *CG5080* expression in *E2f1* mutants to the wild-type, significance is only reached when region within ROI ("front") is quantified, due to the strong *CG5080* signal retained at the back of the CVM migrating collective. For "all" CVM cells, the difference is not statistically significant (ns) (G) unlike when calculated just for change at the front (see Fig. 4G). Two tailed one-way ANOVA with corrections for multiple comparisons using statistical hypothesis testing was performed. Triple asterisks mark $p<0.001$ for comparison between the s*tg*^{4} mutants and wild-type. Scale bar: 50µm.

Development • Supplementary information

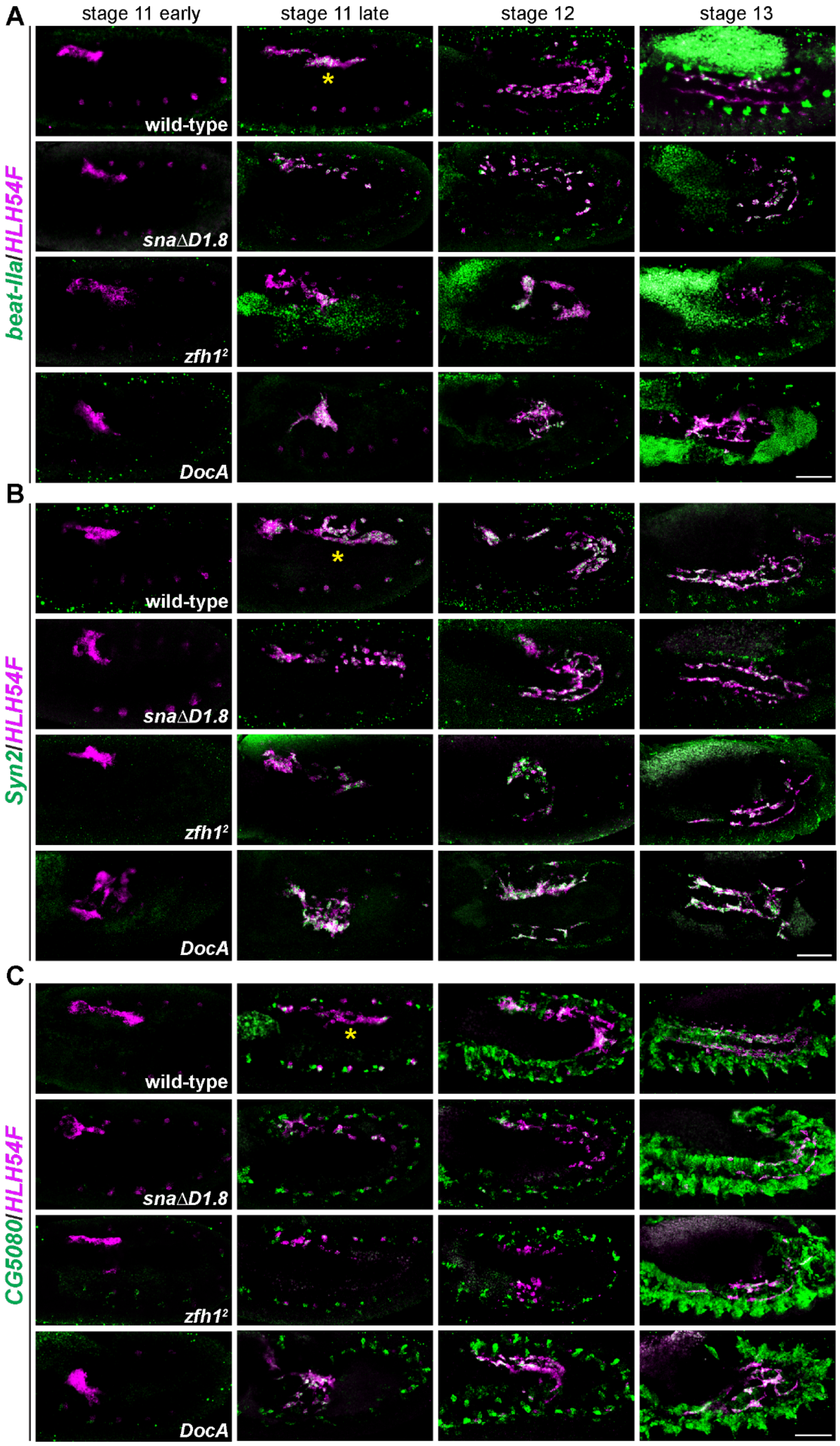

Development • Supplementary information

Fig. S4. The timing of late gene expression in CVM cells is not affected by mutation of *sna*, *zfh1* or *Doc* genes. Normally initiating expression late at stage 11, timing of *beat-IIa* (green, A), *Syn2* (green, B) and *CG5080* (green, C) transcription in the CVM (labeled by *HLH54F* probe in magenta, A-C) is unaffected by loss of *sna*, *zfh1* or *Doc*. *zfh1* mutant embryo stage 11 early in (A, *zfh1*) showing *beatIIa* (depicted here in panel A) was also costained with *Doc2* (not shown here, but depicted in Figure 5F). *snaΔD1.8* mutant embryo stage 11 late in (C, *snaΔD1.8*) showing *CG5080* was also costained with *Doc2* (not shown here but depicted in Figure 5E). *DocA* mutant embryo stages 11 early and late (A, *DocA*) showing *beatIIa* was also costained with *kon* (not shown here but depicted in Figure 6B). Asterisks indicate normal initiation of expression for genes in wild type, that is similar in the mutants. Scale bar: 50µm.

Development • Supplementary information

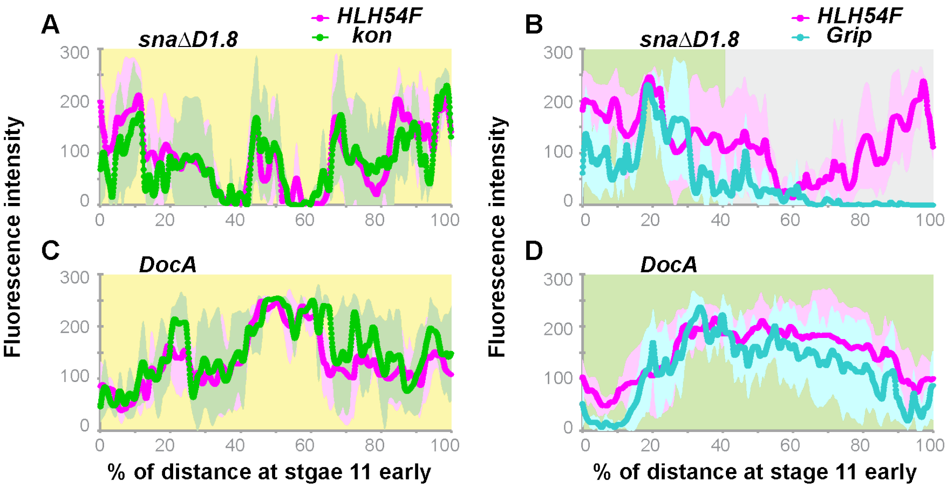

Fig. S5. *sna* and *DocA* mutants exhibit disrupted polarized expression in migrating CVM cells. Quantification of *kon* (A,C) and *Grip* (B,D) expression in the CVM cells as measured by its fluorescence intensity profile from anterior (0%) to posterior (100%) along with *HLH54F* (see Methods). *kon* fails to be enriched in the front CVM cells (yellow area throughout) in both *snaΔD1.8* (A) and *DocA* (C) mutants. *Grip* localization in the back portion of CVM is largely unchanged/normal in *snaΔD1.8* mutants (B) but broadly expressed/abberantin *DocA* mutants as *Grip* is detected in almost all CVM cells (green vs gray area, D). Compare with wildtype in Fig. 6E,G.

Development • Supplementary information

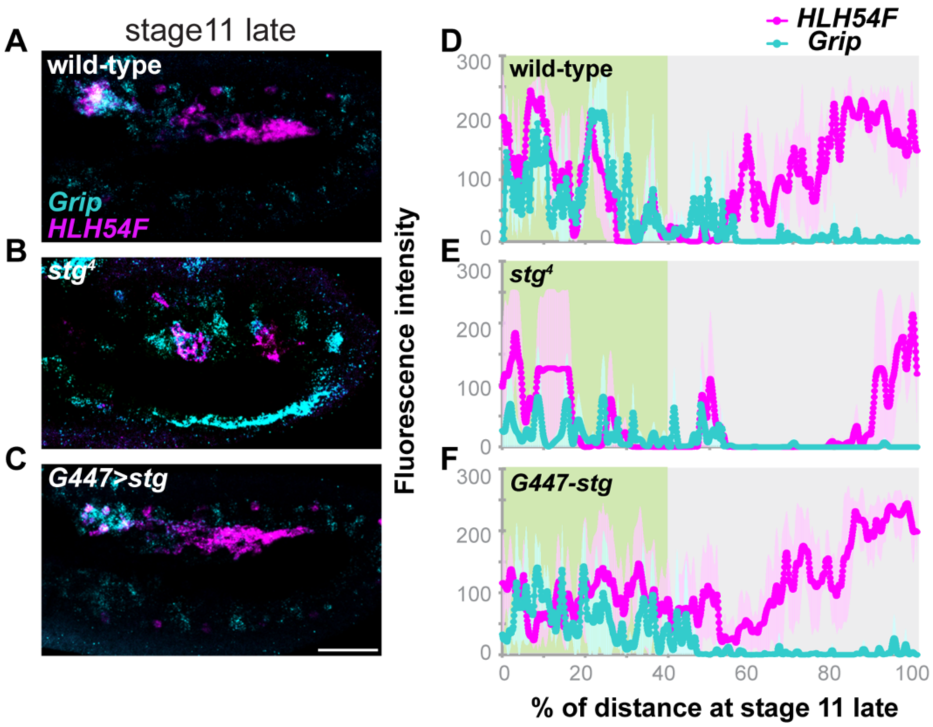

Fig. S6. Disrupting cell cycle progression has little effects on polarized gene expression in the CVM. (A-C) Expression of *Grip* (cyan, A-C) in CVM cells (labeled by *HLH54F* in magenta) detected by in situ hybridization with HCR probes in wild-type and cell cycle mutants. **(D-F)** Quantification of *Grip* localization in the CVM (stage 11 late) as measured by its fluorescence intensity profile from anterior (0%) to posterior (100%) along with *HLH54F* (see Methods). Green shaded areas indicate overlapping between *Grip* and *HLH54F*, while gray shading indicates low-to-no expression.

Table S1. MEME motifs from analysis of 10 enhancer sequences using XSTREME.
Positional weight matrices for *de novo* motifs relating to Figure 3G.

Available for download at
https://journals.biologists.com/dev/article-lookup/doi/10.1242/dev.202262#supplementary-data
