# Supplementary material for: Two sequential gene expression programs bridged by cell division support long-distance collective cell migration
Source: Development. 2024 May 17;151(10):dev202262. doi: 10.1242/dev.202262 (PMC11165717; doi:10.1242/dev.202262)
Supplement: Supplementary file 1 [file develop-151-202262_review_history.pdf]

Two sequential gene expression programs bridged by cell division support long-distance collective cell migration

Jingjing Sun, Ayse Damla Durmaz, Aswini Babu, Frank Macabenta and Angelike Stathopoulos
DOI: 10.1242/dev.202262

Editor: Swathi Arur

Review timeline

Original submission:	14 August 2023
Editorial decision:	16 October 2023
First revision received:	6 March 2024
Editorial decision:	27 March 2024
Second revision received:	28 March 2024
Accepted:	8 April 2024

Original submission

First decision letter

MS ID#: DEVELOP/2023/202262

MS TITLE: Two sequential gene expression programs bridged by cell division support long-distance collective cell migration

AUTHORS: Jingjing Sun, Aswini Babu, Frank Macabenta, and Angelike Stathopoulos

I have now received all the referees' reports on the above manuscript, and have reached a decision. The referees' comments are appended below, or you can access them online: please go to BenchPress and click on the 'Manuscripts with Decisions' queue in the Author Area.

As you will see, the referees express considerable interest in your work, but have some significant recommendations to improve the clarity and the rigor of the analysis. If you are able to revise the manuscript along the lines suggested, which may involve further experiments, I will be happy receive a revised version of the manuscript. Your revised paper will be re-reviewed by one or more of the original referees.

Please attend to all of the reviewers' comments and ensure that you clearly highlight all changes made in the revised manuscript. Please avoid using 'Tracked changes' in Word files as these are lost in PDF conversion. I should be grateful if you would also provide a point-by-point response detailing how you have dealt with the points raised by the reviewers in the 'Response to Reviewers' box. If you do not agree with any of their criticisms or suggestions please explain clearly why this is so.

Reviewer 1

Advance summary and potential significance to field

The manuscript entitled “Two sequential gene expression programs bridged by cell division support long-distance collective cell migration” by Sun et al presents the idea that cell cycle regulation plays an important role in CVM cell migration. They show that there are specific transcription

© 2024. Published by The Company of Biologists under the terms of the Creative Commons Attribution License (https://creativecommons.org/licenses/by/4.0/).

factors that are expressed either continuously throughout CVM migration, only early or only later during CVM migration. They also show that disrupting the cell cycle also can disrupt gene expression in the CVM. This study adds a significant and important dimension to the process of collective cell migration. My main critique of this manuscript is the clarity of the presentation of the data, which I go into with more specifics below.

Comments for the author

Major comments
Line 85-87 and Figure 1B: HLH54F expression is already shown in Figure 1A, and it is very hard to see the expression of zfh1 in Figure 1B. I suggest showing just zfh1 expression in Figure 1B or separate the HLH54F and zfh1 channels in the figure. Additionally, if demonstrating co-localization is the goal, higher magnification images would help more to make your case.

Figure 2: It would be clearer for the reader to show one panel of HLH54F expression (as the top row shows), and the rest of the rows are just the gene indicated because right now the HLH54F expression (magenta) is so strong it is hard to see the exact expression of the other genes. As the figure is now, it looks like every gene is expressed in the CVM at the same level.

Line 159: It would be very helpful for the reader if Pseudotime trajectory analysis was explained and described in general before getting into the specific data. Specifically, why this type of analysis for this study?

Figure 3: UMAP plots need to be explained better because I have no idea how these plots correlate with gene expression at different times during CVM migration. Explanation should be in the text and in the body of the figure. It should be clear exactly what the authors want the reader to get from each piece of data.

Figure 4: The PH3 channel in Figure 4C and 4F needs to be shown separately because it is very hard to see any difference in PH3 expression in mutants compared to wild type. Also, higher magnification images would also help in demonstrating changes in PH3 expression levels.

Figure 5B: Comparing Figure 5B to 5A, it looks like there are more CVM cells in Figure 5B (zfh12 mutant). It could be due to slightly different stages, but this should be clarified.

Line 322-325: The authors state that kon and Grip are no longer differentially localized in DocA mutants, but are broadly expressed in all CVM cells. CVM cells do not migrate properly in DocA mutants, and specifically do not seem to spread anteriorly at all. Is it known whether DocA mutants have the same number of CVM cells as wild type? Have the authors checked for apoptosis in DocA mutants? If there is cell death in DocA mutants, it would be hard to justify the statement that kon and Grip are broadly expressed in all CVM cells. It could also be that the CVM cells are bunched together in DocA mutants so the anterior and posterior CVM cells are all in the same location. In this case, there would be no way of distinguishing purely spatially anterior from posterior cells.

Figure 7: It is very hard to see Doc2 expression in Figure 7I. Two suggestions are to show Doc2 expression separately from HLH54F expression and show a higher magnification image.

Minor comments
Throughout the manuscript, the authors used quotation marks around genotype names – for example Line 201: “CG5953.” The quotation marks are very distracting and make it seem like the genotypes stated are not real because of the quotation marks.

Line 87: Change Figure 1A to Figure 1B

Line 106: Change correspond to corresponding and change Figure 1B to Figure 1C

Line 238: Change (arrows, C, “wild type”) to (arrows, 4C, “wild type”)

Line 250: Change compromised to decreased – compromised is not explicit enough for this statement.

© 2024. Published by The Company of Biologists under the terms of the Creative Commons Attribution License (https://creativecommons.org/licenses/by/4.0/).

Line 257-260: There is no Figure 4I in the figure.

Line 351: Change TF to TFs

Line 352: Change active to actively

Reviewer 2

Advance summary and potential significance to field

This study from Sun et al. examines the caudal visceral mesoderm (CVM) of the Drosophila embryo, which collectively migrate, divide, and migrate again during embryogenesis. The researchers had previously identified a number of genes expressed in these cells through RNAseq, and here used a combination of in situ hybridization, reporter lines that they generated, curated ChIP data, and mutant analysis to characterize spatiotemporal gene expression during migration. They identified three classes of gene expression patterns: continuous, widespread early and restricted, or late. Using this and genomic information, they determined a set of associated binding sites, and mapped some to putative regulators that could mediate the expression patterns.

They further characterize expression of continuous gene HLH54F and late genes syn2 and Cg5080 in wild type and cell cycle mutants. Cg5080 is highly expressed at the time of cell division and when division is forced, and lower in embryos mutant for the E2f1 cell cycle regulator. They go on to show/confirm that mutants for the early genes sna, zfh1, docA have poorer migration and altered expression of several downstream genes, especially ones that are distributed differentially across the A/P axis ("polarized"). They do not see a similar impact on polarized gene expression when cell cycle is blocked. They propose a general model that early genes promote invasion, cell division switches off this program, and then continuous/late factors activate of a set of genes that contribute to differentiation and a polarized pattern of differentiating cells.

Overall this is a data-intensive paper with detailed characterization of several factors important in CVM migration/identity and their regulatory regions. The data is carefully analyzed with attention to detail and is well-described in the text. The mechanism that is proposed about the cell cycle acting as a reset in gene expression is interesting and could be applicable to other developmental cell types. However, this idea is not explored very rigorously, so currently the paper is most relevant to understanding the CVM cell type in detail and general mechanisms of enhancer structure. The impact of the paper would be significantly strengthened with more clear/direct demonstration of mechanism.

Comments for the author

Major concerns-

One of the more novel aspects of the paper is the idea that there is a cell cycle bridge between two transcriptional programs. Given this, the data to support this need to be very clear and quantified. For example, loss of E2F1 function results in apparent changes to late genes Cg5080 and syn2 expression, shown in Figure 4 but these data are difficult to discern from images, especially as overlays. They are also hard to interpret since the number of cells is changing, which would change the level of signal. The results would be clearer and more convincing if they were quantified- eg, what percentage of CVM cells increase/lose cg5080 or syn2 expression when cell cycle is increased/blocked at each embryonic stage? It may also help to show more examples or quantify the extent of Cg5080/syn2 expression in the CVM by cell cycle stage.

In addition, more integration of the E2F1 putative binding site data in the text may be helpful. The binding site data from supplemental figure 4 would be helpful to be added to the enhancers in figure 3H to help tie this aspect of the story together. The authors claim that "matches to the E2f1 binding consensus are enriched within the late enhancers (Figure S3A)." but it is not clear what they mean – enriched relative to what? Figure S3A shows that 3/4ths of the late genes and 2/3rds of

© 2024. Published by The Company of Biologists under the terms of the Creative Commons Attribution License (https://creativecommons.org/licenses/by/4.0/).

the continuous genes have E2F1 binding sites, and given the small numbers, it's not clear that represents enrichment.

The authors propose the model that there are two sequential gene expression programs in the CVM, but it seems like there could alternatively be a gradation of changing set of factors to refine gene expression since several factors seem to be needed early and remain on at later times too (eg, they aren't all switched off by the cell cycle). The late requirement for zfh1, sna, and DocA is tested for late expression patterns of HLH45F and beatIIa (supplemental figure 4, discussed on lines 300- 301), but it is clear that different factors may regulate each expressed gene. Thus, the authors need to test their hypothesis further by examining the effect of these early mutants (zfh1, sna, and DocA loss of function) on the key late genes they identify – Cg5080 and Syn2 – to better support the conclusion there are two separate programs (and not an accumulation of factors, or that the situation just differs for each gene). The other evidence the authors use to suggest there are two programs is the data shown in Figure 7J/K; when Doc2 is on, Cg5080 is off, and vice versa; this is also importantly linked to changes in cell cycle progression. But again it is hard to know how widespread this correlation is from only a couple of images – some quantification would be very helpful. As a key part of the main idea of the paper, more clarity is important here.

Minor
This manuscript relies on several pieces of data that comes from an unpublished manuscript (Sun et al., in revision), which makes it difficult to evaluate the impact and contribution of this paper over the other. Is the other paper available?

There is a lot of data here, but it isn't always clear how it fits together. So at times the paper is hard to digest and it feels like there are some jumps in the logic. Several new CVM genes are described, and then enhancer analysis identified five key regulators for these, but then mutant analysis is conducted primarily on other genes, not the key regulators that were identified, so it does not feel like it all is part of the same story. For the mutant analysis, it may make more sense to re-order the figures to present early defects (figure 5) first, then the cell cycle (figure 4), and then late stage defects.

Zfh1 is identified in the continuous class, and the most extensive mutant characterization is completed for this gene, and the connection to the enhancers is shown in figure 1. It is predicted to regulate HLH54F, kon, syn among others and the manuscript shows that mutants for zfh1 have lower Doc expression and expanded domains of kon and Grip expression in the CVM. Given this, it may make sense to include the data from Suppl fig 2 A and E on Grip in the main paper to provide more consistency and increase the focus on this characterization.

In fig 1C, what defines the non-Zfh1 binding enriched areas? Zfh1 seems high in much of the region of CG5080, for example.

Line 178 asserts that byn expression in a UMAP profile "suggests that it likely acts prior to the active migration" because it is only detected early- however given this is RNA and not protein analysis, it is not entirely fair to make this conclusion.

Figure 3D is small, so the colors of the boxes (especially green/teal) are hard to distinguish, and could use a scale to show enhancer sizes. It would also be useful to include the sites of predicted Zfh1 (and E2F) binding here.

At the bottom of page 15-top of 16, the authors postulate that the different severity of phenotypes in the different mutants may be linked to different levels of polarity retained in the collective. This seems very speculative, especially since many of these mutants affect multiple tissues in the developing embryo which may also contribute to the severity of phenotypes. The idea could be more directly tested, given the tools the authors have generated, and they should try this or de-emphasize this interpretation in the test. For example, they could reduce cell numbers with CVM-Gal4 and cell death or cell cycle regulators to test the idea of a "minimum" collective that they postulate about, or express "polarity" genes in a larger or smaller subset of CVM cells to examine impact on movement.

© 2024. Published by The Company of Biologists under the terms of the Creative Commons Attribution License (https://creativecommons.org/licenses/by/4.0/).

The authors might consider reorganizing figure 7 and its legend to emphasize the key link to changes in the expression programs and link to cell cycle- eg, focus more on Fig 7J/K and perhaps move some of the other data to supplemental.

First revision

Author response to reviewers' comments

Response to reviewers - Sun et al.

Reviewer 1 Advance Summary and Potential Significance to Field:
The manuscript entitled "Two sequential gene expression programs bridged by cell division support long- distance collective cell migration" by Sun et al presents the idea that cell cycle regulation plays an important role in CVM cell migration. They show that there are specific transcription factors that are expressed either continuously throughout CVM migration, only early or only later during CVM migration. They also show that disrupting the cell cycle also can disrupt gene expression in the CVM. This study adds a significant and important dimension to the process of collective cell migration. My main critique of this manuscript is the clarity of the presentation of the data, which I go into with more specifics below.

Reviewer 1 Comments for the Author:
Major comments

Line 85-87 and Figure 1B: HLH54F expression is already shown in Figure 1A, and it is very hard to see the expression of zfh1 in Figure 1B. I suggest showing just zfh1 expression in Figure 1B or separate the HLH54F and zfh1 channels in the figure. Additionally, if demonstrating co-localization is the goal, higher magnification images would help more to make your case.
We updated Figure 1 and added a higher magnification view of *zfh1* and *HLH54F* in situs. We feel this works the best to show the co-expression of these two TFs. We felt that showing *zfh1* expression alone would not be as helpful since there is a lot of expression in somatic muscles, making *zfh1* expression in the CVM cells hard to discern.

Figure 2: It would be clearer for the reader to show one panel of HLH54F expression (as the top row shows), and the rest of the rows are just the gene indicated because right now the HLH54F expression (magenta) is so strong it is hard to see the exact expression of the other genes. As the figure is now, it looks like every gene is expressed in the CVM at the same level.
We agree with the reviewer that in some cases it is hard to see the green signals and assess the expression levels of the other genes. In fact, levels from certain genes (i.e., *mew*, *sna*, *CG5080*) are high in the surrounding tissues, while signals in the CVM are really low in comparison and difficult to demonstrate convincingly. However, data from different types of experiment such as the enhancer results in Fig. 1 and the single-cell RNAseq in Fig. 3 all provide supporting evidence that these genes are indeed expressed in the CVM. We tried only showing the green, however, we still feel the magenta HLH54F signals are very helpful in identifying the cells and the overlapping white signal are easier to see (we provide one example below; *HLH54F*, *mew*, and *zfh1* were costained however showing *mew* and *zfh1* without the *HLH54F* reference for CVM is hard to interpret). For these reasons, we decided not to update Fig. 2.

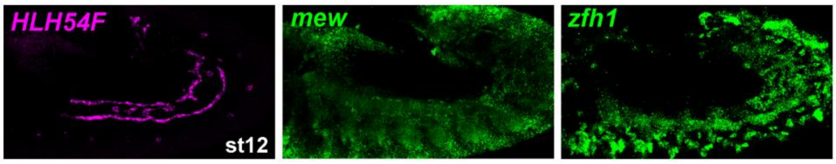

Line 159: It would be very helpful for the reader if Pseudotime trajectory analysis was explained and described in general before getting into the specific data. Specifically, why this type of analysis for this study?
We updated the text to explain pseudotime analysis, which helps to reinforce the gene expression

© 2024. Published by The Company of Biologists under the terms of the Creative Commons Attribution License (https://creativecommons.org/licenses/by/4.0/).

dynamics identified using in situ hybridization and HCR probes.

Figure 3: UMAP plots need to be explained better because I have no idea how these plots correlate with gene expression at different times during CVM migration. Explanation should be in the text and in the body of the figure. It should be clear exactly what the authors want the reader to get from each piece of data.
We updated the text to explain UMAP plots, and how cluster 10 relates specifically to CVM cells. UMAP plots for individual genes allow us to make inferences about timing of expression within the CVM (coupled with pseudotime trajectory analysis).

Figure 4: The PH3 channel in Figure 4C and 4F needs to be shown separately because it is very hard to see any difference in PH3 expression in mutants compared to wild type. Also, higher magnification images would also help in demonstrating changes in PH3 expression levels.
We added higher magnification images to increase visibility and also split the channels for *CG5080* expression versus PH3 stainings (see Fig. 4G). However, as discussed above, we decided to show merged channels for the other stainings as it's very difficult to interpret without the CVM reference staining (GFP or HLH54f). We feel the signals from HLH54F/GFP are necessary to locate the CVM cells, so that one knows where to look for PH3 signals, which are everywhere and even more prominent in the surrounding tissues.

Figure 5B: Comparing Figure 5B to 5A, it looks like there are more CVM cells in Figure 5B (zfh12 mutant). It could be due to slightly different stages, but this should be clarified.
In the figure legend, we clarified that Fig. 5B stage 11 image is slightly older in *zfh1* mutant than wild type. It is therefore even more meaningful that *sna* expression perdures in the mutant.

Line 322-325: The authors state that kon and Grip are no longer differentially localized in DocA mutants, but are broadly expressed in all CVM cells. CVM cells do not migrate properly in DocA mutants, and specifically do not seem to spread anteriorly at all. Is it known whether DocA mutants have the same number of CVM cells as wild type? Have the authors checked for apoptosis in DocA mutants? If there is cell death in DocA mutants, it would be hard to justify the statement that kon and Grip are broadly expressed in all CVM cells.

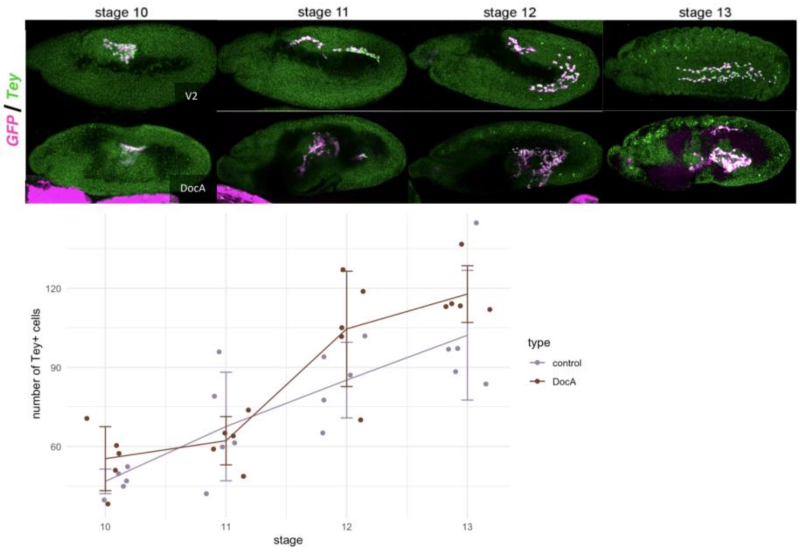

We assayed *DocA* mutants for cell death, we counted number of Tey+ and/or GFP+ CVM cells over time in embryos that carry the HLH54F-venus transgene, and did not find a significant difference between number of cells present in control vs *DocA* homozygous mutants over time (stages 10-13; see the quantification graph). When cells die, they retain GFP but lose Tey signal. Number of CVM cells increases in control and *DocA* mutants in similar manner. Therefore, we do not believe that cell death contributes to the loss of polarity within the collective, as cell number in *DocA* mutants is comparable to wildtype.

It could also be that the CVM cells are bunched together in DocA mutants so the anterior and

© 2024. Published by The Company of Biologists under the terms of the Creative Commons Attribution License (https://creativecommons.org/licenses/by/4.0/).

posterior CVM cells are all in the same location. In this case, there would be no way of distinguishing purely spatially anterior from posterior cells.

While the *DocA* mutants do exhibit aberrant morphology (i.e. more clumpy), we detect expanded and overlapping *kon* and *Grip* expression at all stages and even in single plane confocal scans. We speculate Doc is important to maintain polarity and repress the late gene expression program. As *kon* and *Grip* are expressed broadly at stage 13 (after cells' divide), it is also possible that the expanded *kon* and *Grip* expression domains detected in *DocA* mutants is a result of turning on the late gene expression program, early.

Figure 7: It is very hard to see Doc2 expression in Figure 7I. Two suggestions are to show Doc2 expression separately from HLH54F expression and show a higher magnification image.
We have updated Fig. 7 to show magnified views of *Doc2* and *HLH54F* (see Fig. 7D). We did not show expressions separately because we felt it was not as helpful to the reader. However, we did quantify the expression changes (see Fig. 7E).

Minor comments

Throughout the manuscript, the authors used quotation marks around genotype names – for example Line 201: “CG5953.” The quotation marks are very distracting and make it seem like the genotypes stated are not real because of the quotation marks.
The quotation marks were removed from genotypes.

Line 87: Change Figure 1A to Figure 1B
Corrected.

Line 106: Change correspond to corresponding and change Figure 1B to Figure 1C
Corrected.

Line 238: Change (arrows, C, “wild type”) to (arrows, 4C, “wild type”)
Corrected.

Line 250: Change compromised to decreased – compromised is not explicit enough for this statement.
Corrected.

Line 257-260: There is no Figure 4I in the figure.
Corrected - changed to Fig. 4E.

Line 351: Change TF to TFs
Corrected.

Line 352: Change active to actively
Corrected.

***** Reviewer 2 Advance Summary and Potential Significance to Field:
This study from Sun et al. examines the caudal visceral mesoderm (CVM) of the Drosophila embryo, which collectively migrate, divide, and migrate again during embryogenesis. The researchers had previously identified a number of genes expressed in these cells through RNAseq, and here used a combination of in situ hybridization, reporter lines that they generated, curated ChIP data, and mutant analysis to characterize spatiotemporal gene expression during migration. They identified three classes of gene expression patterns: continuous, widespread early and restricted, or late. Using this and genomic information, they determined a set of associated binding sites, and mapped some to putative regulators that could mediate the expression patterns.

They further characterize expression of continuous gene HLH54F and late genes syn2 and Cg5080 in wild type and cell cycle mutants. Cg5080 is highly expressed at the time of cell division and when

© 2024. Published by The Company of Biologists under the terms of the Creative Commons Attribution License (https://creativecommons.org/licenses/by/4.0/).

division is forced, and lower in embryos mutant for the E2f1 cell cycle regulator. They go on to show/confirm that mutants for the early genes sna, zfh1, docA have poorer migration and altered expression of several downstream genes, especially ones that are distributed differentially across the A/P axis ("polarized"). They do not see a similar impact on polarized gene expression when cell cycle is blocked. They propose a general model that early genes promote invasion, cell division switches off this program, and then continuous/late factors activate of a set of genes that contribute to differentiation and a polarized pattern of differentiating cells.

Overall this is a data-intensive paper with detailed characterization of several factors important in CVM migration/identity and their regulatory regions. The data is carefully analyzed with attention to detail and is well-described in the text. The mechanism that is proposed about the cell cycle acting as a reset in gene expression is interesting and could be applicable to other developmental cell types. However, this idea is not explored very rigorously, so currently the paper is most relevant to understanding the CVM cell type in detail and general mechanisms of enhancer structure. The impact of the paper would be significantly strengthened with more clear/direct demonstration of mechanism.

Reviewer 2 Comments for the Author:
Major concerns-

One of the more novel aspects of the paper is the idea that there is a cell cycle bridge between two transcriptional programs. Given this, the data to support this need to be very clear and quantified. For example, loss of E2F1 function results in apparent changes to late genes Cg5080 and syn2 expression, shown in Figure 4, but these data are difficult to discern from images, especially as overlays. They are also hard to interpret since the number of cells is changing, which would change the level of signal. The results would be clearer and more convincing if they were quantified- eg, what percentage of CVM cells increase/lose cg5080 or syn2 expression when cell cycle is increased/blocked at each embryonic stage? It may also help to show more examples or quantify the extent of Cg5080/syn2 expression in the CVM by cell cycle stage.
We expanded Fig. 4 to include quantification of CG5080 and Syn2 expressions when cell proliferation is decreased (*stg*) or increased (i.e. G447>stg) (see Fig. 4F,G).
We added a quantification of CG5080 vs PH3 signal finding that the presence of both is correlated (Fig. 4I). We also expanded Fig. 7 to include quantification of *CG5080* expression in the CVM at various stages (st11 early - stage 13) when cell proliferation is increased (i.e. 5053>stg) in comparison to wildtype (see Fig. 7E), and when Doc2 is overexpressed (i.e. 5053>Doc2) (see Fig. 7G).

In addition, more integration of the E2F1 putative binding site data in the text may be helpful. The binding site data from supplemental figure 4 would be helpful to be added to the enhancers in figure 3H to help tie this aspect of the story together. The authors claim that "matches to the E2f1 binding consensus are enriched within the late enhancers (Figure S3A)." but it is not clear what they mean – enriched relative to what? Figure S3A shows that 3/4ths of the late genes and 2/3rds of the continuous genes have E2F1 binding sites, and given the small numbers, it's not clear that represents enrichment.
We understand the reviewer's concern and changed this statement to read "matches to the E2f1 binding consensus are present within the late enhancers and absent from early enhancers (Fig. S3A)". For most genes, we kept the E2f1 binding site matches within enhancers in the supplement (Fig. S3) but did move the CG5080 enhancer with E2f1 binding site matches to the main figure (Fig. 4K).

The authors propose the model that there are two sequential gene expression programs in the CVM, but it seems like there could alternatively be a gradation of changing set of factors to refine gene expression since several factors seem to be needed early and remain on at later times too (eg, they aren't all switched off by the cell cycle). The late requirement for zfh1, sna, and DocA is tested for late expression patterns of HLH45F and beatIIa (supplemental figure 4, discussed on lines 300- 301), but it is clear that different factors may regulate each expressed gene. Thus, the authors need to test their hypothesis further by examining the effect of these early mutants (zfh1, sna, and DocA loss of function) on the key late genes they identify – Cg5080 and Syn2 – to better support the conclusion there are two separate programs (and not an accumulation of factors, or

© 2024. Published by The Company of Biologists under the terms of the Creative Commons Attribution License (https://creativecommons.org/licenses/by/4.0/).

that the situation just differs for each gene).
We understand the reviewer's concern and agree that each of the late genes may be differentially regulated. We show that early mutants (*zfh1*, *sna* and *DocA* loss of function) have little effect on timing of expression for *beatIIa*, *Syn2*, or *CG5080* expression (Fig. S4). We added the new data for *Syn2* to revised Fig. S4; like *beatIIa*, there was no change on *Syn2* expression in these mutants.

The other evidence the authors use to suggest there are two programs is the data shown in Figure 7J/K; when Doc2 is on, Cg5080 is off, and vice versa; this is also importantly linked to changes in cell cycle progression. But again it is hard to know how widespread this correlation is from only a couple of images – some quantification would be very helpful. As a key part of the main idea of the paper, more clarity is important here.
We examined more embryos and strengthened the evidence that *Doc2* and *Cg5080* expression is mutually exclusive by adding quantitative analysis (see Fig. 7E). These data show that as Doc2 levels are reduced within the migrating collective, that *CG5080* levels simultaneously increase; exhibiting opposite trends (Fig. 7E). The transition is faster when *stg* is overexpressed.
In addition, we added more support for the repressive effect of Doc2 on *CG5080*. *CG5080* appears to be positively regulated by cell cycle progression and yet also repressed upon ectopic expression of Doc2 (Fig. 7F,G).
We also added new data in which we assayed the effect of overexpression of *stg* in the *DocA* mutant background; our rationale was that perhaps two requirements must be met in order to transition to the late gene expression program. Indeed, we found that this results in strong precocious *CG5080* expression that is observable early at stage 11 (Fig. 7H,I).

Minor
This manuscript relies on several pieces of data that comes from an unpublished manuscript (Sun et al., in revision), which makes it difficult to evaluate the impact and contribution of this paper over the other. Is the other paper available?
Yes - this study is published now in Cell Reports (Sun et al. *Cell Reports* Oct 2023. PMCID:PMC10704487). In this previous study, we produced the scRNA-seq data, UMAP, and trajectory analysis that led to the finding that *Doc* is expressed at the front of the CVM migrating collective. In this prior study, we did not comment on any late gene expression in the CVM, namely *Syn2*, *CG5080*, and *beatIIa* genes.

There is a lot of data here, but it isn't always clear how it fits together. So at times the paper is hard to digest and it feels like there are some jumps in the logic. Several new CVM genes are described, and then enhancer analysis identified five key regulators for these, but then mutant analysis is conducted primarily on other genes, not the key regulators that were identified, so it does not feel like it all is part of the same story. For the mutant analysis, it may make more sense to re-order the figures to present early defects (figure 5) first, then the cell cycle (figure 4), and then late stage defects.
The idea we were trying to get across was that a standard analysis of enhancers did not turn up any obvious insights into temporal dynamics so we needed to think more about the behavior of cells as they migrate. It was key to us to realize that the early and late genes do not overlap and that cells divide and lose spatial patterning. We have worked to present the data in the way we think best showcases this story and also have worked to increase clarity of the message in this revised manuscript.

Zfh1 is identified in the continuous class, and the most extensive mutant characterization is completed for this gene, and the connection to the enhancers is shown in figure 1. It is predicted to regulate HLH54F, kon, syn among others and the manuscript shows that mutants for zfh1 have lower Doc expression and expanded domains of kon and Grip expression in the CVM. Given this, it may make sense to include the data from Suppl fig 2 A and E on Grip in the main paper to provide more consistency and increase the focus on this characterization.
The mutually-exclusive spatial expression domains of *kon* and *Grip* were already published in our previous paper (Sun et al. *Cell Reports* Oct 2023). We do now emphasize the *kon* and *Grip* expression patterns at the top of Fig. 6; we added panels showing individual *kon* and *Grip* expression domains.

In fig 1C, what defines the non-Zfh1 binding enriched areas? Zfh1 seems high in much of the region of CG5080, for example.

© 2024. Published by The Company of Biologists under the terms of the Creative Commons Attribution License (https://creativecommons.org/licenses/by/4.0/).

We are not sure, but the reviewer's question led us to investigate if Zfh1 might repress CG5080. However, overexpression of *zfh1* in the CVM does not repress CG5080. We are tight on space and chose not to include this negative result.

Line 178 asserts that byn expression in a UMAP profile "suggests that it likely acts prior to the active migration" because it is only detected early- however, given this is RNA and not protein analysis, it is not entirely fair to make this conclusion.
We agree. While we had mentioned the possibility that the Byn protein product perdures, we further clarified this section to make sure that this point comes across.

Figure 3D is small, so the colors of the boxes (especially green/teal) are hard to distinguish, and could use a scale to show enhancer sizes. It would also be useful to include the sites of predicted Zfh1 (and E2F) binding here.
We added a scale to show enhancer size, but did not include Zfh1 or E2fF1 binding sites as the point of this figure is to show the sites identified by computational approaches (XSTREME). E2fF1 is relevant later after making the link to cell cycle; we emphasize the matches to the E2fF1 binding consensus in Fig. S3 and added the CG5080 enhancer with E2fF1 binding sites highlighted to Fig. 4K.

At the bottom of page 15-top of 16, the authors postulate that the different severity of phenotypes in the different mutants may be linked to different levels of polarity retained in the collective. This seems very speculative, especially since many of these mutants affect multiple tissues in the developing embryo, which may also contribute to the severity of phenotypes. The idea could be more directly tested, given the tools the authors have generated, and they should try this or de-emphasize this interpretation in the test. For example, they could reduce cell numbers with CVM-Gal4 and cell death or cell cycle regulators to test the idea of a "minimum" collective that they postulate about, or express "polarity" genes in a larger or smaller subset of CVM cells to examine impact on movement.
We have deemphasized this point.

The authors might consider reorganizing figure 7 and its legend to emphasize the key link to changes in the expression programs and link to cell cycle- eg, focus more on Fig 7J/K and perhaps move some of the other data to supplemental.

We agree and have added quantitative analysis to Fig 7 and expanded on the mutually exclusive relationship of *CG5080* and *Doc2*; some of the data for *Grip* was moved to new Fig. S6.

In particular, we point out that we added a quantitative analysis for CG5080 expression over a timecourse (Fig. 7E), and upon ectopic expression of string (Fig. 7E) or Doc2 (Fig. 7G).

Furthermore, we were pleased to find that ectopic expression of *string* in a *DocA* mutant background leads to widespread precocious expression of *CG5080* in the migrating CVM collective. This supports the view that cell cycle is a positive input and *Doc* genes are repressive inputs.

Second decision letter

MS ID#: DEVELOP/2023/202262

MS TITLE: Two sequential gene expression programs bridged by cell division support long-distance collective cell migration

AUTHORS: Jingjing Sun, Ayse Damla Durmaz, Aswini Babu, Frank Macabenta, and Angelike Stathopoulos

I have now received all the referees reports on the above manuscript, and have reached a decision. The referees' comments are appended below, or you can access them online: please go to BenchPress and click on the 'Manuscripts with Decisions' queue in the Author Area.

© 2024. Published by The Company of Biologists under the terms of the Creative Commons Attribution License (https://creativecommons.org/licenses/by/4.0/).

The overall evaluation is positive and we would like to publish a revised manuscript in Development. However, Reviewer 2 makes some suggestions to further improve the clarity of the work. I do not expect to send the manuscript back to the reviewer but instead will look at the incorporation of the suggestions myself. Thus, please attend to all of the reviewers' comments in your revised manuscript and detail them in your point-by-point response.

Reviewer 1

Advance summary and potential significance to field

This revised manuscript by Sun et al. thoroughly describes gene expression changes that occur throughout the migration of the caudal visceral mesoderm (CVM) during Drosophila embryogenesis. Furthermore, the authors examine the regulatory mechanisms involved in these gene expression patterns from early to late cell migration. The advancement of the field is significant because it brings out this idea of distinct temporal groupings during morphogenesis, which is not necessarily a new concept, but it has been shown here in an elegant way.

Comments for the author

I am satisfied with the current revisions and fully support acceptance of this manuscript in its current revised form.

Reviewer 2

Advance summary and potential significance to field

The manuscript by Sun et al. explores the idea that distinct gene expression profiles in the migratory caudal visceral mesoderm (CVM) of the Drosophila embryo are divided by a cell division cycle, and that the divisions are important for resetting the expression patterns. This is an interesting concept, and multiple cell types, such as neural crest, do need to divide in the course of their migration, so the findings may be applicable to gene expression regulation in other cell types. It may also be relevant to metastatic cancer cells as they lose control of the cell cycle. Overall, the work in rigorous, detailed, and comes to interesting conclusions that will be of broad interest.

Comments for the author

The manuscript has improved in revisions and is now more convincing and clear. My concerns have all been addressed, although I have some very minor suggestions below.

Minor suggestions:

I think the quantitative analysis on the gene expression overlap is very helpful (Fig 4 F, G, Fig 7 E, G, I) and makes a stronger case for the arguments. I am curious, though, how this was done and why it is called “gene exp. size” on the graphs. Is it simply the percentage of cells that have both markers, or does it somehow reflect the spatial domain or size of the regions that are expressing both markers?

For Fig 6I, what is the genotype of these embryos? In particular, what is driving the GFP expression? I understand the mutants have multiple defects, but I wasn’t sure I could see the CVM cells veering off course as described in the text (page 16), especially if there are more cells, although the Doc2 mutants look off course. Perhaps the authors could point to particular defects in these images.

Second revision

Author response to reviewers' comments

© 2024. Published by The Company of Biologists under the terms of the Creative Commons Attribution License (https://creativecommons.org/licenses/by/4.0/).

Minor suggestions:
I think the quantitative analysis on the gene expression overlap is very helpful (Fig 4 F, G, Fig 7 E, G, I) and makes a stronger case for the arguments. I am curious, though, how this was done and why it is called “gene exp. size” on the graphs. Is it simply the percentage of cells that have both markers, or does it somehow reflect the spatial domain or size of the regions that are expressing both markers?
As the HCR probes localize to cytoplasm, and even with highly specific HLH54F probes, the imaging resolution does not allow us to count the number of cells, the best we could do is to calculate the relative spatial domains. So yes, the reviewer is correct, the quantification reflects the spatial domain /size of the regions that express both markers.
We updated the y-axis labels from "relative expression size" to "relative expression domain", which might work better to facilitate understanding. We also included a few sentences to explain what was done in the quantification.

For Fig 6I, what is the genotype of these embryos? In particular, what is driving the GFP expression? I understand the mutants have multiple defects, but I wasn’t sure I could see the CVM cells veering off course as described in the text (page 16), especially if there are more cells, although the Doc2 mutants look off course. Perhaps the authors could point to particular defects in these images.
We updated the genotype labels to include the V2 transgene and indicated that the anti-GFP is detecting Venus protein driven by an HLH54F enhancer.
We also added an arrow pointing to the migration defect in zfh1 mutants and added comments in the figure legends.

Third decision letter

MS ID#: DEVELOP/2023/202262

MS TITLE: Two sequential gene expression programs bridged by cell division support long-distance collective cell migration

AUTHORS: Jingjing Sun, Ayse Damla Durmaz, Aswini Babu, Frank Macabenta, and Angelike Stathopoulos
ARTICLE TYPE: Research Article

I am happy to tell you that your manuscript has been accepted for publication in Development, pending our standard ethics checks.

© 2024. Published by The Company of Biologists under the terms of the Creative Commons Attribution License (https://creativecommons.org/licenses/by/4.0/).
